# Integrating particle tracking with computational fluid dynamics to assess haemodynamic perturbation by coronary artery stents

**DOI:** 10.1371/journal.pone.0271469

**Published:** 2022-07-28

**Authors:** Luke Boldock, Amanda Inzoli, Silvia Bonardelli, Sarah Hsiao, Alberto Marzo, Andrew Narracott, Julian Gunn, Gabriele Dubini, Claudio Chiastra, Ian Halliday, Paul D. Morris, Paul C. Evans, Perrault C. M.

**Affiliations:** 1 Department of Mechanical Engineering, University of Sheffield, Sheffield, United Kingdom; 2 INSIGNEO Institute, University of Sheffield, Sheffield, United Kingdom; 3 Laboratory of Biological Structure Mechanics–LaBS, Department of Chemistry, Materials and Chemical Engineering ‘Giulio Natta’, Politecnico di Milano, Milan, Italy; 4 Department of Infection, Immunity and Cardiovascular Disease, University of Sheffield, Sheffield, United Kingdom; 5 Department of Mechanical and Aerospace Engineering, Politecnico di Torino, Turin, Italy; 6 Eden Microfluidics, Paris, France; University of Birmingham, UNITED KINGDOM

## Abstract

**Aims:**

Coronary artery stents have profound effects on arterial function by altering fluid flow mass transport and wall shear stress. We developed a new integrated methodology to analyse the effects of stents on mass transport and shear stress to inform the design of haemodynamically-favourable stents.

**Methods and results:**

Stents were deployed in model vessels followed by tracking of fluorescent particles under flow. Parallel analyses involved high-resolution micro-computed tomography scanning followed by computational fluid dynamics simulations to assess wall shear stress distribution. Several stent designs were analysed to assess whether the workflow was robust for diverse strut geometries. Stents had striking effects on fluid flow streamlines, flow separation or funnelling, and the accumulation of particles at areas of complex geometry that were tightly coupled to stent shape. CFD analysis revealed that stents had a major influence on wall shear stress magnitude, direction and distribution and this was highly sensitive to geometry.

**Conclusions:**

Integration of particle tracking with CFD allows assessment of fluid flow and shear stress in stented arteries in unprecedented detail. Deleterious flow perturbations, such as accumulation of particles at struts and non-physiological shear stress, were highly sensitive to individual stent geometry. Novel designs for stents should be tested for mass transport and shear stress which are important effectors of vascular health and repair.

## Introduction

Percutaneous coronary intervention and stent implantation is performed in over three million patients each year to treat coronary artery stenosis [1]. Thrombosis and in-stent restenosis are major adverse consequences of stent deployment [2, 3]. They are induced by penetration of the coronary artery wall to expose sub-endothelial, pro-thrombotic and pro-coagulant factors to circulating blood [4, 5]. In addition, the denudation of the endothelium during stent deployment leads to the loss of local protective effects of endothelial cells, such as production of anti-coagulants and suppression of thrombosis [6, 7]. The design of the stent also has an impact upon thrombosis: stent type, strut thickness, cell size and configuration, the amount of exposed metal and its composition are important factors in post-intervention vascular health [8–14].

Blood flow patterns vary through the arterial tree according to differences in anatomy and pulsatility and are critical regulators of vascular physiology and function [15–18]. We and others demonstrated that stents cause perturbations in flow that prevent vascular cell repair and can therefore increase the risk of in-stent restenosis and thrombosis [7, 19]. Flow disturbances alter the transport of cells, molecules and gases from the bloodstream to the arterial wall and also modify shear stress which alters endothelial function [20, 21]. The influence of stent struts on local shear stress has been studied previously [22–25], however the possible effects on mass transport to the arterial wall is complex and has not been analysed. To address this, we developed a novel experimental platform integrating *in vitro* particle tracking with computational fluid dynamics (CFD) to compare the effects of multiple stent designs upon fluid dynamics.

We aimed to create an experimental platform through which the biomechanical conditions of stented vessels could be studied, by developing a unique combination of *in vitro* and *in silico* techniques.

## Materials and methods

### Assessing stent geometry

Stent struts were measured *in situ* via scaled microscope imaging. The orientation of struts relative to the direction of flow was measured using the directionality function in the Fiji ImageJ plug-in (National Institutes of Health). The software returned a table of structure orientation (angle relative to 0°, the direction of flow), and the length of structure at each orientation as a percentage of the total strut length within each stent (S1 Fig).

### PDMS vessels and stent deployment

2.5 mm diameter [26, 27] *in vitro* model vessels were fabricated using Polydimethylsiloxane [28] (PDMS, Sylgard 184, Dow Corning Corp.; S2 Fig). A range of coronary stents (S1 and S2 Tables) were deployed following clinical guidelines and manufacturers’ instructions, using a 1.2:1 balloon to vessel ratio [11]. Correct stent deployment was confirmed by visual inspection under a light microscope (AE2000, Motic).

### Defining flow rates and entrance length in PMDS vessels

The volumetric flow rate required to attain 1Pa wall shear stress (physiological wall shear stress in arteries) was calculated using the Hagen-Poiseuille equation: *τ_mean_* = 4*μQ*/(*πr*^3^)

*Where*, *Q* is the volumetric flow rate, *τ_mean_* the mean wall shear stress, *r* the vessel radius and *μ* the dynamic viscosity of the fluid.

Mean velocity, *V*, was calculated by dividing flow rate by cross-sectional area: *V* = *Q*/(*πr*^2^)

The Reynolds number (*Re*) could then be calculated, using vessel diameter *D* and fluid density *ρ*: *Re* = *VDρ*/*μ*

Finally, the Reynolds number was used to calculate the entrance length (*L*) required for laminar flow with a parabolic velocity profile to fully develop within the vessel: *L* = EL*D

*EL* is the entrance length number, which is dependent on the flow regime. Assuming a transition between laminar and turbulent flow at *Re* = 2300: for laminar flow, the accepted correlation is: *E*(*laminar*) = 0.06*Re*.

The above was calculated for vessels using viscosity and density data for the full range of fluids used to allow suitable flow rates and entrance lengths to be used.

### Particle tracking

Vessels were connected to a fluid reservoir and peristaltic pump and perfused with cell culture medium (DMEM) containing 10 μm polystyrene particles (1.44 x 10^4^ particles/ml; FluoSpheres, Fisher Scientific) at a flow rate required to generate a Reynolds number of 68, equivalent to that in blood flow of 1 Pa wall shear stress. After a minimum period of 2 minutes, allowing particles to distribute uniformly throughout the fluid, image acquisition was carried out using an inverted microscope and connected camera (AE2000 and Moticam 2300, Motic). The fluid velocity was 12.88 ml/min and imaging was at 20 frames/sec which ensured that particles could be imaged multiple times in the same field of view and therefore amenable to tracking. The bottom-most struts were brought into focus in order to observe particle motion local to the stent and vessel wall, within a depth of field equivalent to 1–1.5x strut depth (depending on the individual stent). Data (two 30-second videos) were captured at overlapping regions along the length of each stent, ensuring complete coverage. Each 30-second video recorded the flow of 50% of the fluid reservoir’s total volume. The acquisition was performed at different regions of the stent in order to capture features that are representative of the entire stent geometry.

The motion of particles between consecutive frames was tracked and analysed using ImageJ (S3 Fig). The orientation of particle tracks relative to the direction of flow was calculated. Multiple particles following common tracks were counted and their distribution was quantified by noting the position of tracks, and the number of particles moving along them, in relation to a 100 μm grid of 1.5 mm height x 2.5 mm length in each field of view. This information was used to create heat maps, to visualise distribution and areas of particle dispersal or concentration (S3 Fig).

The trapping and accumulation of particles within the stent structure was recorded by flushing the vessel with particle-free fluid before imaging the model on a stereo microscope (SMZ-171 BLED, Motic), under fluorescent light provided by a Stereo Microscope Fluorescence Adapter (NIGHTSEA). All remaining particles within stents and 2 mm up and downstream of them were counted.

### Micro-computed tomography (μCT)

μCT scanning of PDMS casts of stented vessels was performed to reconstruct *in vitro* model geometry *in silico* with a SkyScan 1172 high-resolution desktop μCT scanner (Bruker). Reconstruction and visualisation software (NRecon package and DataViewer, Bruker), was used to convert the projection images of the sample into a series of cross-sectional slices in the vessels’ transaxial plane.

### Meshing and computational fluid dynamic simulation

Reconstructed image datasets were imported into ScanIP software (Synopsys Inc.) and the fluid domain selected via thresholding to form a mask. Following a mesh sensitivity analysis, masks were converted to volume meshes consisting of ≥2 million tetrahedral elements via the ScanIP +FE Module, in FLUENT CFD Output format, using the +FE Free mesh algorithm. Element size and internal volume change rate were altered on a 100-point scale, where element edge length was dependent on voxel size, a product of the original μCT scan resolution. Once complete, the Mesh Quality Inspection Tool was checked for errors or warnings, before mesh files were exported.

Mesh files were imported into ANSYS FLUENT software (ANSYS, Inc.) to simulate the 3D steady-state laminar flow of incompressible Newtonian fluid via the solution of the governing equations. The first is the continuity equation, representing conservation of mass, where *v* is the velocity vector: ∇.*ν* = 0

The second is the Navier-Stokes equation, representing conservation of momentum, where *ρ* is fluid density, *p* pressure and *μ* fluid dynamic viscosity: (*ν*.∇*ν*) = −∇*p*+*μ*∇2*ν*

Cell culture media was modelled with a dynamic viscosity of 0.0008 Pa.s and density of 1005 kg/m3. Blood was modelled with a dynamic viscosity of 0.0035 Pa.s and density of 1060 kg/m3 [23, 29]. No-slip boundary conditions were applied at the wall, which was assumed to be rigid. A gauge pressure of zero was applied at the outlet and a fully developed 3D parabolic flow, of sufficient rate to generate 1 Pa wall shear stress, was applied at the inlet boundary via a user defined function (UDF). Simulation results were assessed using ANSYS CFD-Post (ANSYS, Inc.). Velocity field volume renders and vector plots were displayed to visualise flow around stent struts. Contour plots of wall shear stress on the vessel wall and stent struts were displayed, and the data extracted to find minimum, maximum and average values for vessels. Reference lines along the wall were selected and used to plot changing wall shear stress magnitude through the length of stents.

## Results

The purpose of the study was to develop a workflow to assess haemodynamic perturbation by stents. Several stent designs were analysed to assess whether our methodology was robust across diverse strut geometries.

### Establishing a platform for fluid dynamic assessment

Coronary stents were successfully deployed within 2.5 mm diameter PDMS model vessels, with lumen expansion and strut penetration comparable to *ex vivo* histological samples. 10 μm FluoSphere particles were tracked through model vessels under flow. Under these conditions, particles could be clearly identified and tracked adjacent to the vessel wall and stent struts where velocity was reduced. As a control, particle tracking in empty tubes gave streamlines which were parallel to flow. Tracks were evenly distributed across the vessel, as was the frequency of particles moving along them (S4 Fig).

**Fig 1:**
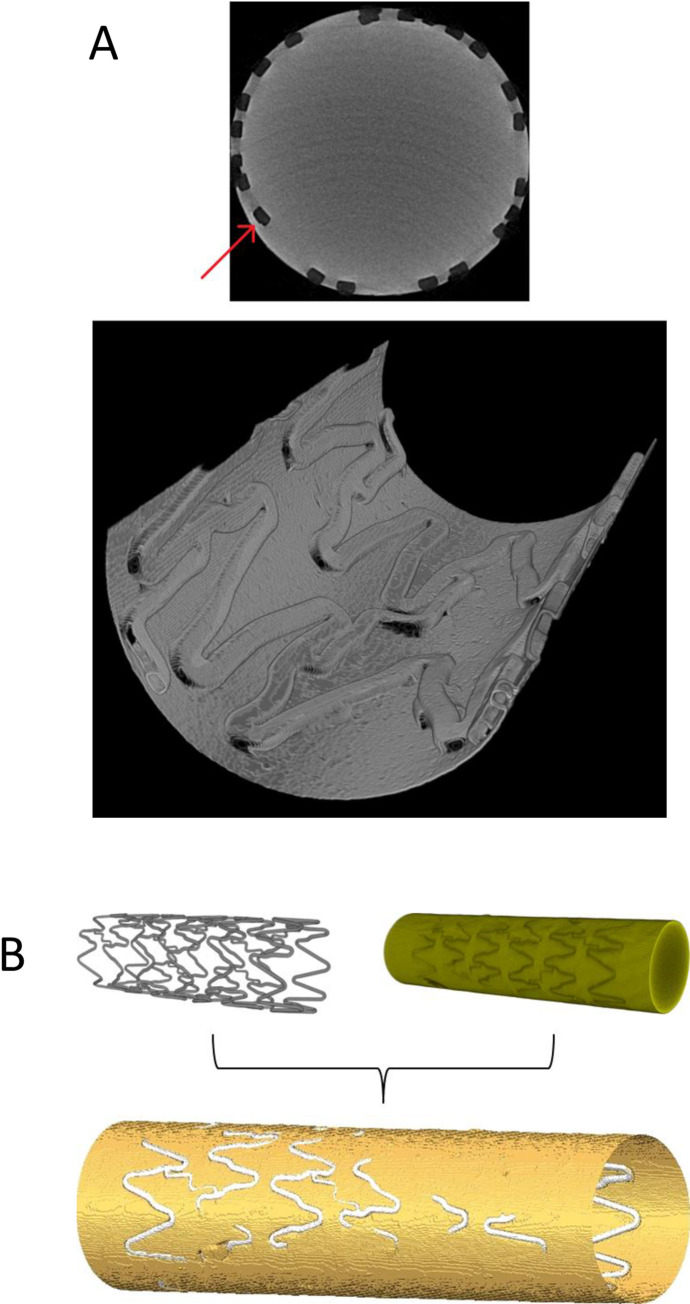
Reconstructed μCT data of a stent cast and registration. **A:** Theuse of PDMS casts of model vessels provided μCT scan data of accurate geometry and captured fine stent strut detail, including prolapsed struts (red arrow). **Top:** Optimising μCT scan and reconstruction parameters for homogeneous PDMS casts produced an image of the lumen with a clearly defined wall boundary. **Bottom:** Cutaway of a section of wall boundary reconstructed from a 9.92 μm resolution scan of a Coroflex Blue coronary stent cast. **B:** Rigid body registration was performed on models of a Coroflex Blue coronary stent and its associated cast, reconstructed from data from separate μCT scans. The two were aligned in an effort to measure the ability of casts to recreate the geometry of stents.

We observed that μCT scans of stents had poor image quality due to metal artefacts from the struts. We therefore generated negative PDMS casts of stented vessels for scanning which allowed visualisation of fine structural details (S5 Fig). This process resulted in accurate reproductions of both stent and vessel wall geometry, allowing preparation of accurate *in silico* models for meshing (Fig 1A). This was confirmed via well-aligned rigid body registration of *in silico* models of stented vessels and their resultant casts (performed in Amira, FEI) (Fig 1B). Meshing and FLUENT set-up variables were informed by residual analysis and mesh sensitivity studies. Stability of the model was achieved with convergence criterion of 10^−6^ and a mesh of ≥2 million elements.

### Particle tracking revealed that coronary stent geometries have major effects on local haemodynamics

**Fig 2:**
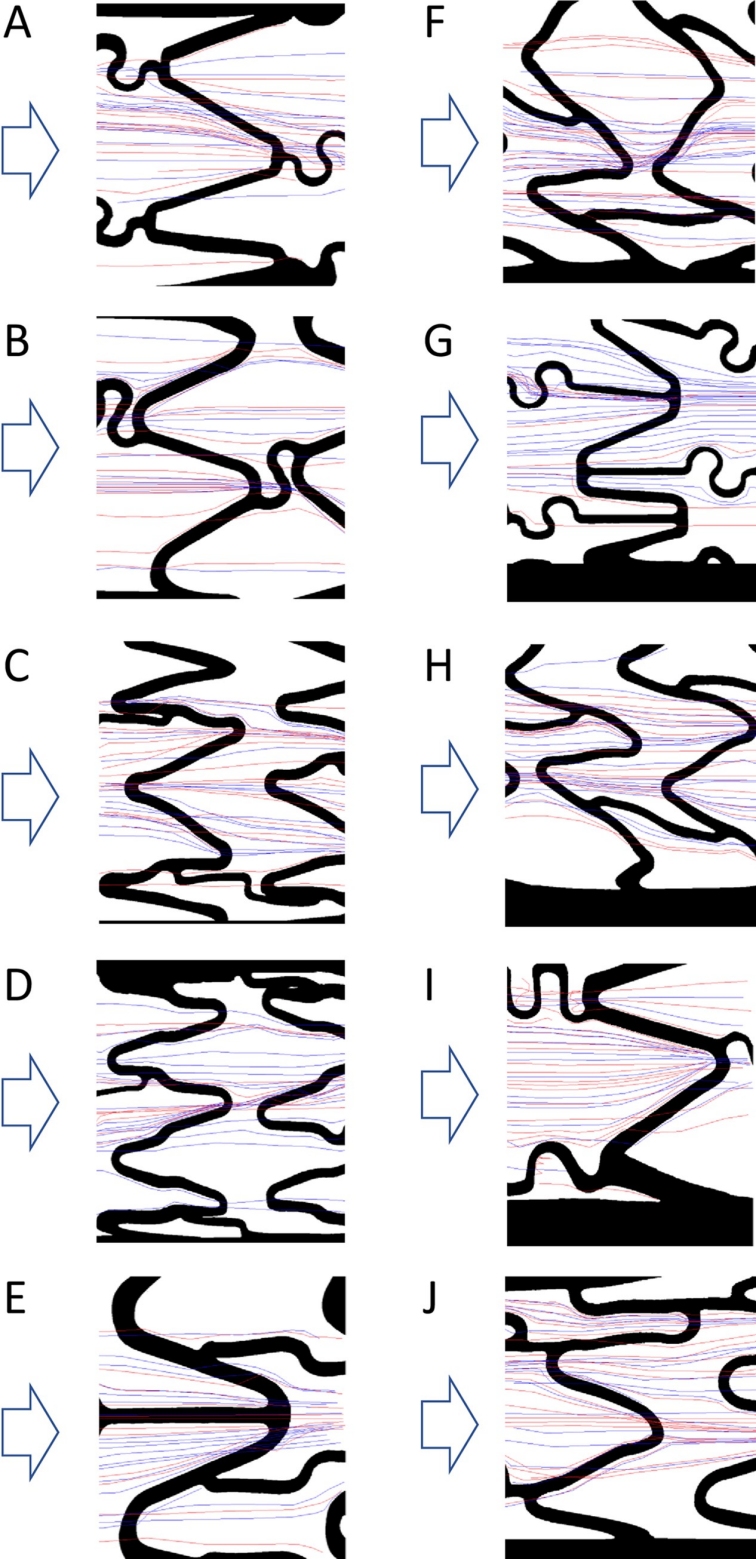
Tracked particle streamlines in coronary stents. Particles were tracked moving through coronary stents deployed within model vessels. Tracking was performed for two 30-second-long sequences (red and blue tracks) on two stent faces, with the stent rotated by 90° between the two faces. **A:** BiodivYsio OC stent; **B:** Chroma stent; **C:** Coroflex Blue stent; **D:** Coroflex Blue Neo stent; **E:** Matrix stent; **F:** Orsiro stent; **G:** Penchant stent; **H:** Pro Kinetic Energy stent; **I:** Velocity stent; **J:** XTRM-Track stent. Flow direction indicated with arrows. Re = 68 (equivalent to blood flow with 1 Pa wall shear stress). Representative images are mid-stent length and the plane of focus on the bottom of the vessel.

**Fig 3:**
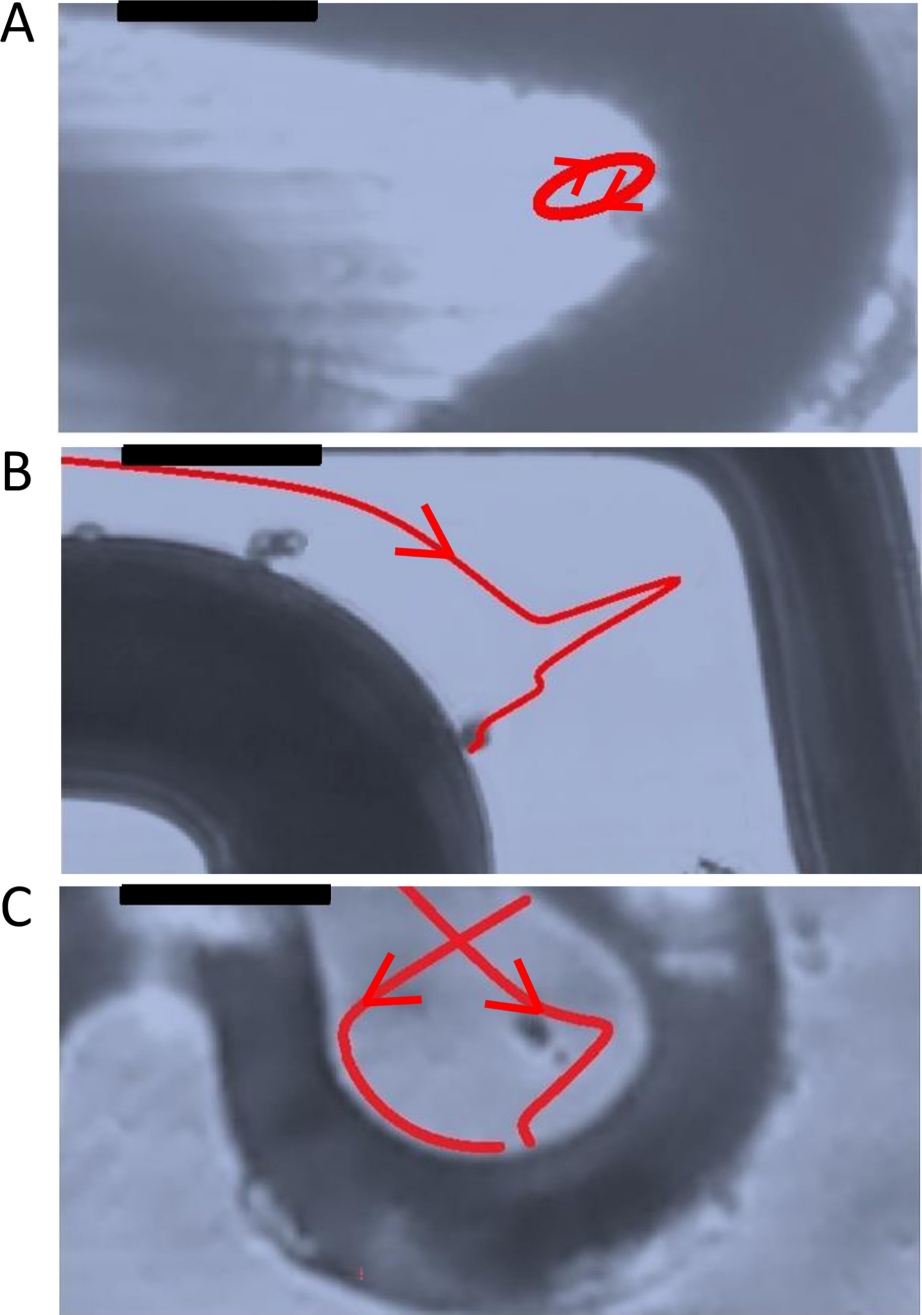
Complex flow within coronary stent geometry. Particle tracking revealed areas of complex flow patterns local to struts within coronary stent geometry. **A:** Recirculation seen within a valley structure of a Coroflex Blue stent. **B:** Flow reversal seen between Coroflex Blue main ring and bridge struts. **C:** Flow reversal and recirculation seen within a bridge strut of a BiodivYsio OC stent. Flow from left to right, Re = 68 (equivalent to blood flow with 1 Pa wall shear stress). Scale bar: 100 μm.

Ten different coronary stent designs were analysed. All were of the laser-cut ‘slotted tube’ type, with struts arranged in a series of circumferential rings connected by bridges. All stent struts had square cross-sections and 90° edge angle and showed a wide range of distinct strut sizes, strut orientation and metal surface area (S1 and S2 Tables; S6 Fig). Particle tracking revealed the impact of the presence of coronary stents on local flow patterns and the disparity between stent designs. Streamlines through coronary stents are shown in Fig 2. There was good agreement between data from two videos taken in each field of view and clear streamline patterns followed from one field of view to the next. In sections of complex stent strut geometry and immediately up and downstream of struts, areas of reversed flow, recirculation and stagnation were identified (Figs 2G, 2I and 3). Stents were seen to have a large effect on track orientation, as particles would typically divert to follow struts for some distance before crossing them and returning to a longitudinal path. Particles tended to follow common streamlines and were funnelled or concentrated by the peak and valley pattern of each ring of struts (S7 Fig).

**Fig 4:**
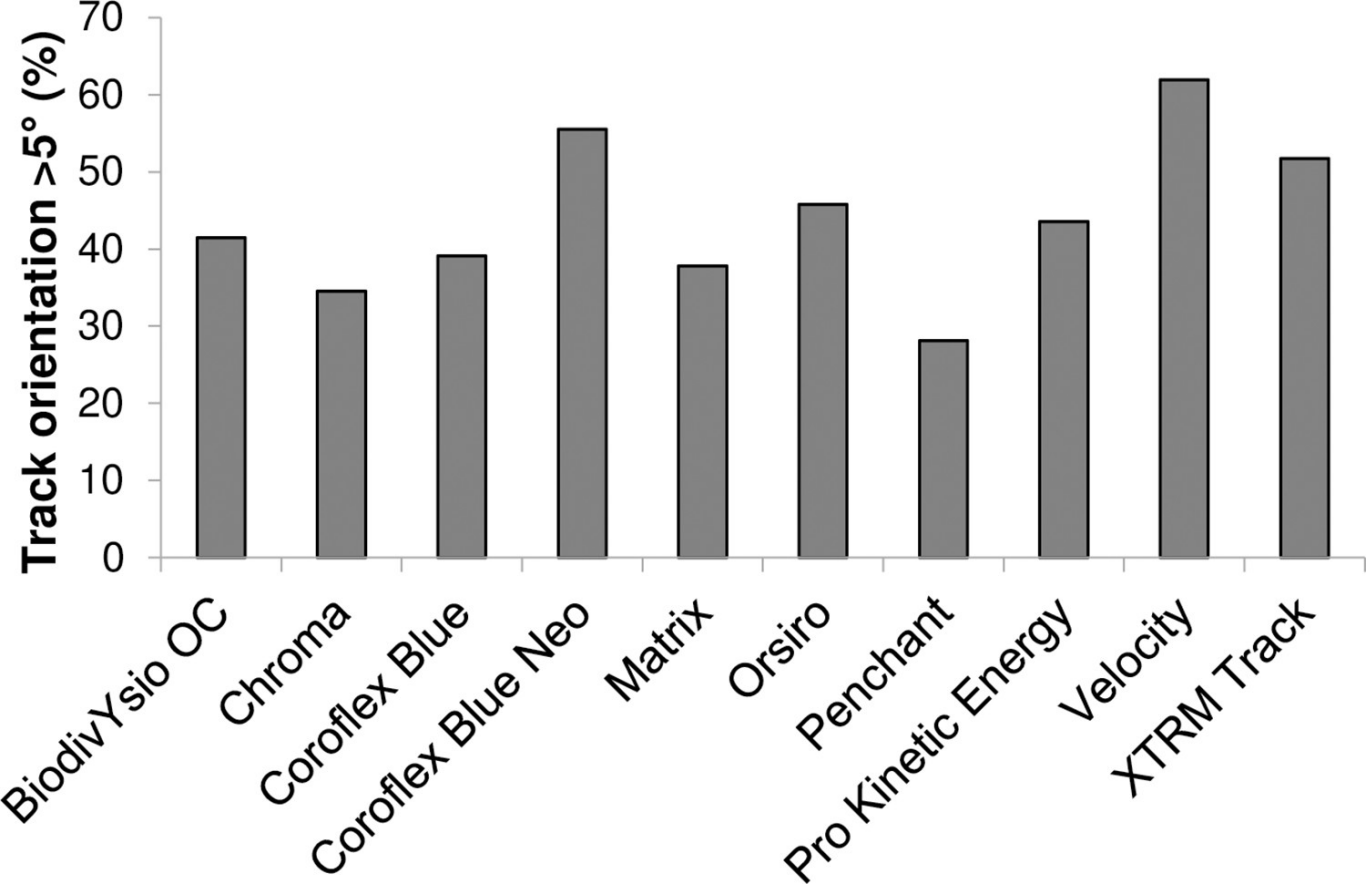
Particle track orientation in coronary stents. The orientationof tracked particle streamlines moving through coronary stents deployed within model vessels was measured to determine whether the workflow can assess the impact of stent design on the direction of flow. The length of tracks at an angle greater than ±5° of the direction of flow (0°) is shown as a percentage of the total length of tracks within each stent. This threshold was applied to ensure that stent-induced flow deviation was captured while minor deviations, due to equipment disturbance or manual tracking errors, were omitted.

**Fig 5:**
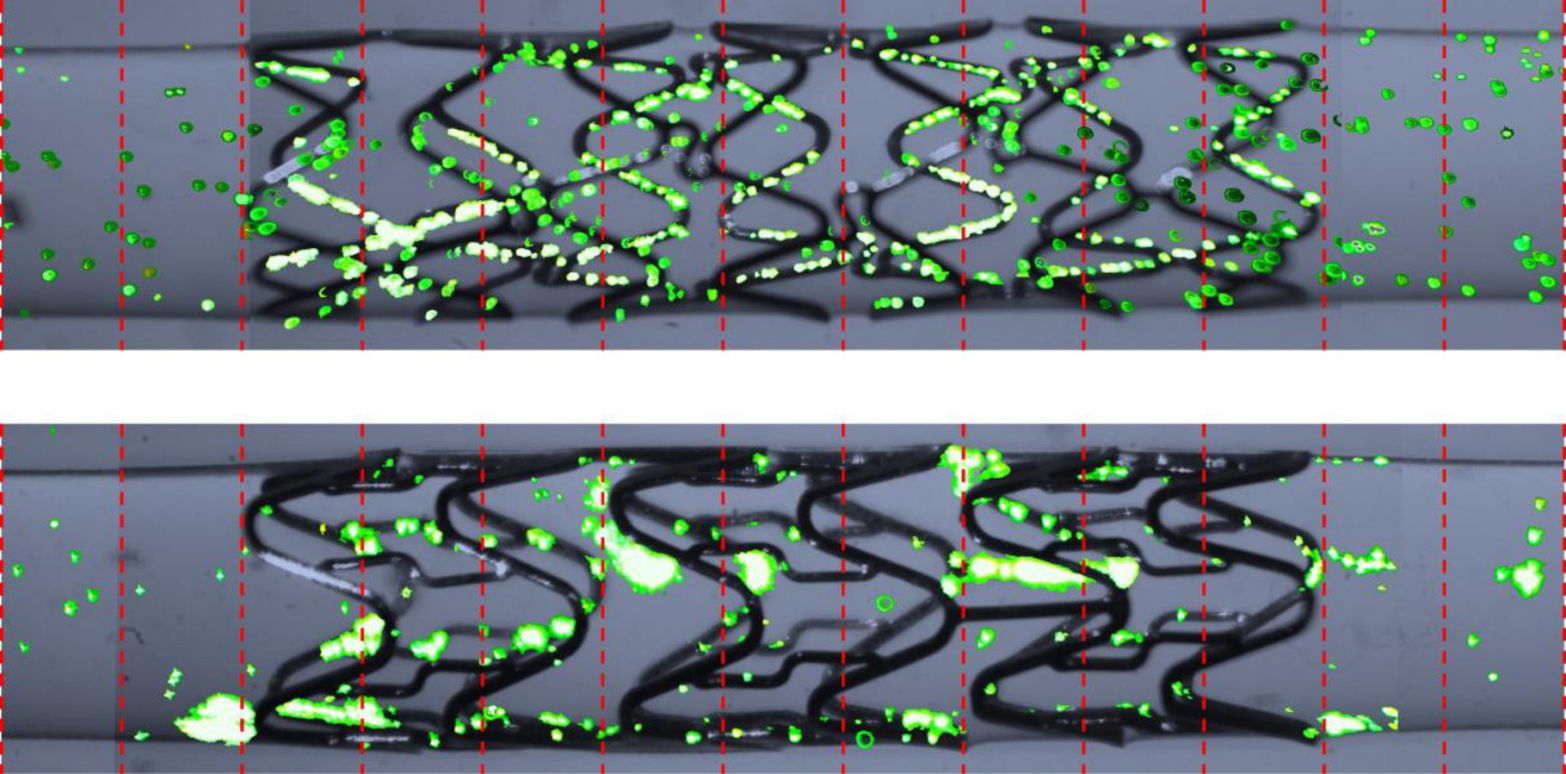
Locations of particle accumulation within coronary stents. After cessation of flow, accumulated particles (green) within coronary stents deployed in model vessels were imaged and counted within 1 mm segments (red lines) in order to assess whether the workflow can be used to analyse particle accumulation. No pattern in the location or distribution of particles throughout each model was seen, with the exception of the Chroma and Matrix stents shown here, in which accumulation was almost exclusively seen immediately up or downstream of struts. **Top:** Chroma stent. **Bottom:** Matrix stent.

To quantify the effects of specific stent geometries upon flow direction we assessed the percentage of tracks that deviated from a path within ±5° of the direction of applied flow (Fig 4). Although flow varied with geometry, there was no clear relationship between the particles’ direction of travel and strut directionality or thickness (S8 Fig). Stent geometry also influenced the sites of particle accumulation in some designs i.e. the Chroma and Matrix stents in which particles almost exclusively settled immediately up or downstream of struts (Fig 5).

### Computational modelling

**Fig 6:**
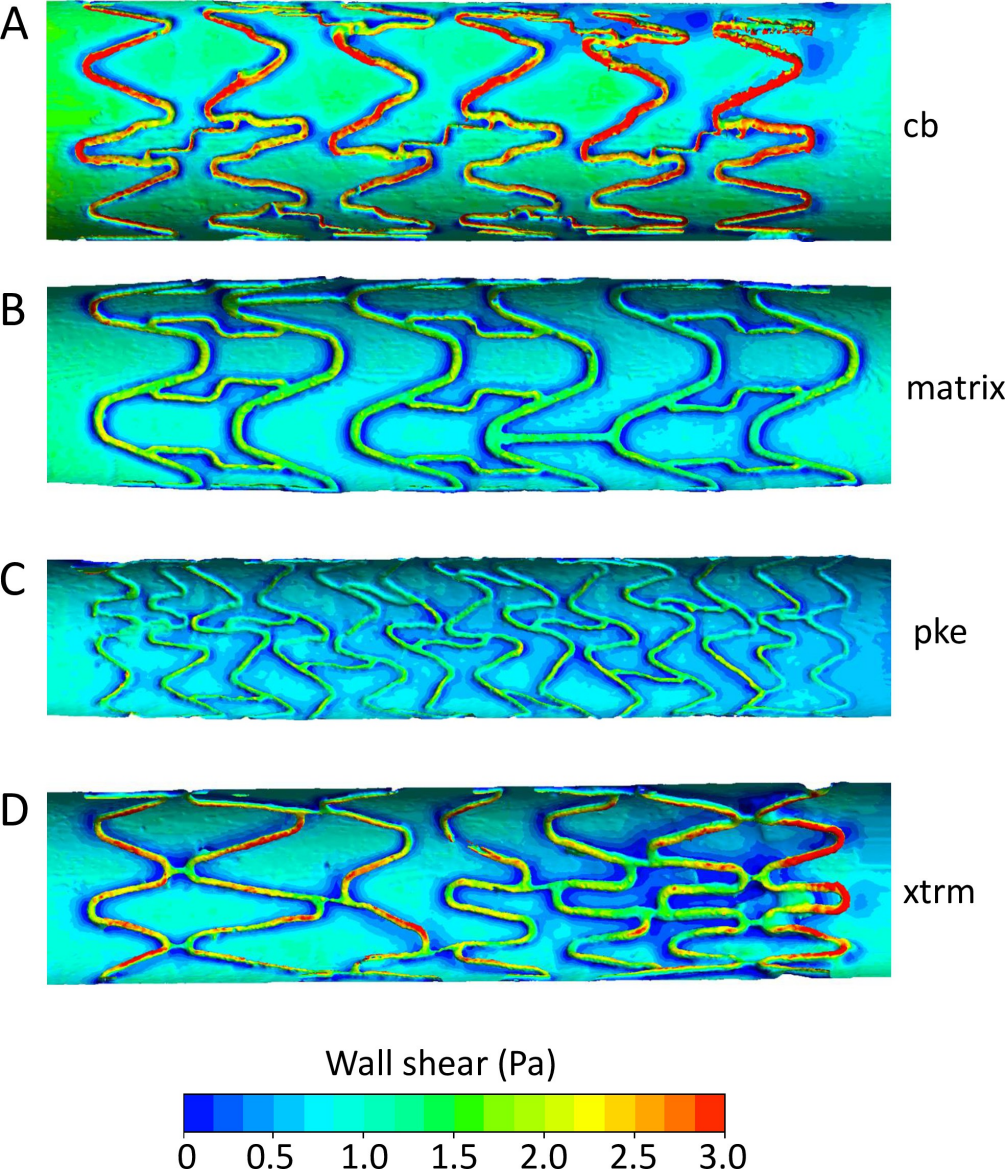
Wall shear stress within coronary stents. *In silico* models were created from reconstructed μCT scans of coronary stent casts and used for CFD analysis to plot wall shear stress along the vessel. **A:** Coroflex Blue stent, showing elevated shear stress on the luminal surface of prolapsed struts; **B:** Matrix stent, showing reduced shear stress downstream of struts; **C:** Pro Kinetic Energy stent, showing reduced shear stress downstream of struts; **D:** XTRM-Track stent, showing reduced shear stress downstream of struts and within areas of complex geometry. Flow from left to right, 1 Pa average wall shear stress. Note: the maximum value of the scale bar is set to 3 Pa to better illustrate regions of low flow.

**Fig 7:**
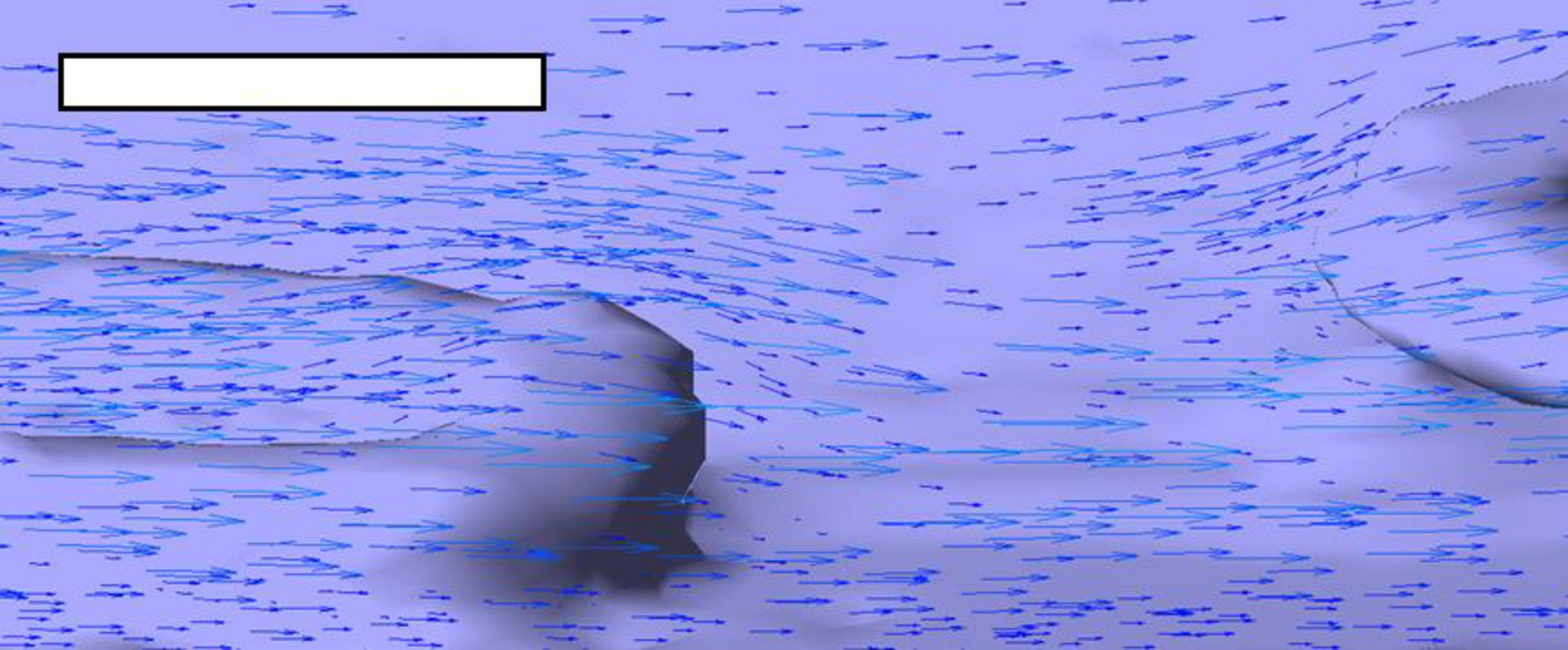
Velocity vectors around coronary stent struts. *In silico* models were reconstructed from Coroflex Blue stent strut μCT data and used for CFD analysis. Velocity fields are depicted at the vessel wall [dark blue arrows] and at a fluid layer positioned nearer to the lumen [light blue arrows], with the length of each arrow being proportional to velocity. Vectors revealed that while stent struts had relatively modest effects on velocity magnitude, they greatly impacted direction as flow moved up and over struts, leading to reduced wall shear stress in those regions. Flow from left to right, 1 Pa average wall shear stress. Scale bar: 100 μm.

Casts of vessels containing Coroflex Blue, Matrix, Pro Kinetic Energy, and XTRM-Track stents were scanned by μCT at near-identical resolution (10 μm ±2 μm). Fully developed blood flow of 1 Pa wall shear stress was simulated within the resultant CFD models. The presence of coronary stents greatly altered the pattern of wall shear stress distribution and magnitude (Fig 6). Quantitative measures of the area-weighted average wall shear stress (AWA-WSS) showed reduction to 0.79 Pa in the XTRM-Track, 0.78 Pa in the Matrix, and 0.65 in the Pro Kinetic stents. In the Coroflex Blue, AWA-WSS was skewed by high shear forces on the luminal surface of struts that were detached from the luminal surface (Fig 6A). In this example, struts contributed to an AWA-WSS of 1.07 Pa. However, shear stress on the wall below these protruding struts was, in places, reduced to as low as 0.05 Pa. There was agreement between particle tracking and CFD velocity profiles, e.g. funnelling by the peak and valley patterns of struts (S9 Fig). Stents had little influence upon velocity magnitude, but did alter the direction of flow up and over struts (Fig 7). This reduced the longitudinal component of velocity and thus also reduced wall shear stress. All coronary stents displayed regions of wall shear stress magnitude <0.5 Pa. These regions were focal to bridges and areas where struts from adjacent rings were in close proximity, hindering downstream recovery of longitudinal velocity (Fig 7). Immediately downstream of ‘valleys’ in circumferential strut rings, shear stress was reduced to as low as 0.1 Pa. It should be noted that the influence of struts on velocity was restricted to highly localised layers of fluid nearest to the wall whereas more luminal layers were unaffected (Fig 7; compare velocity fields at the vessel wall [dark blue arrows] versus those nearer to the lumen [light blue arrows]).

**Fig 8:**
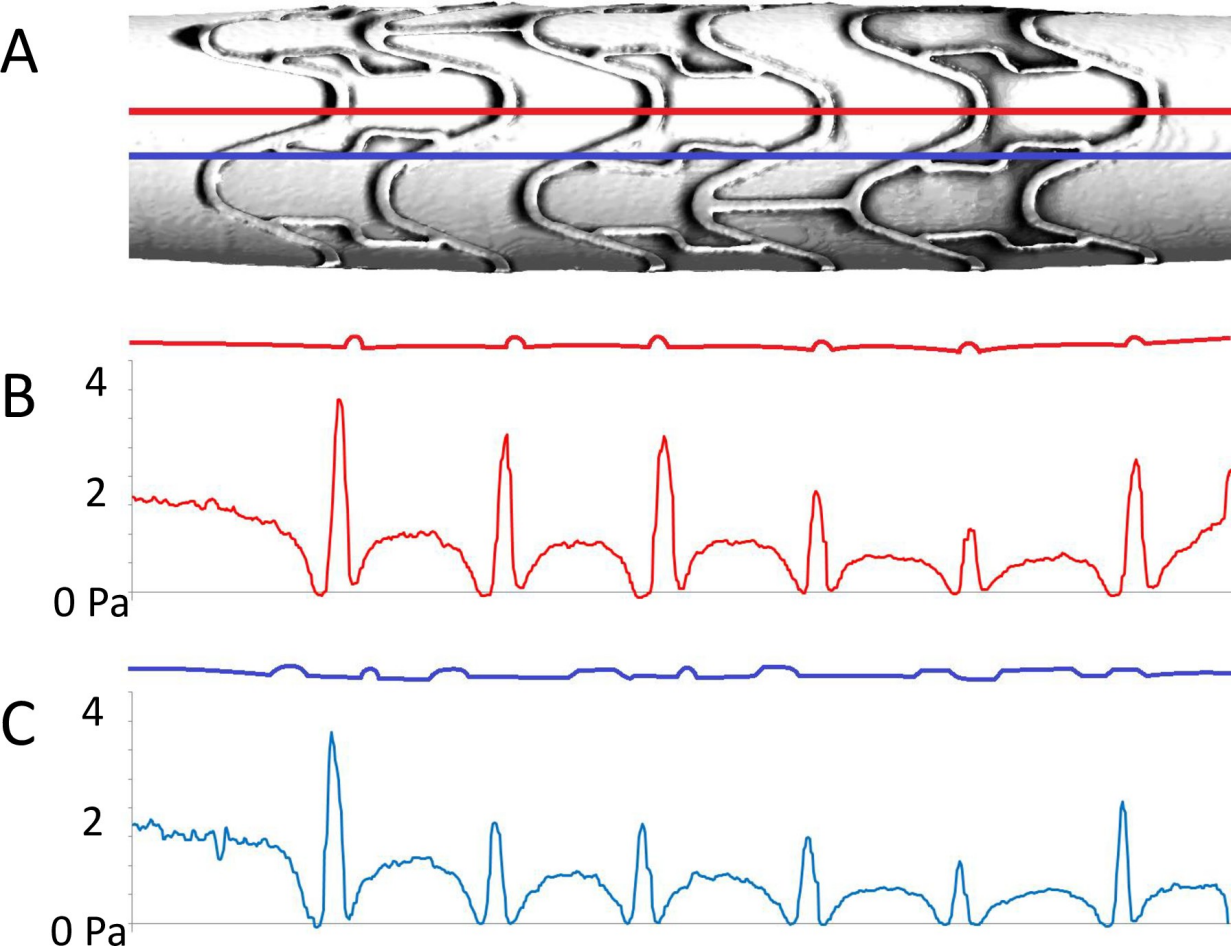
Wall shear stress along reference lines within a Matrix coronary stent. *In silico* models were reconstructed from coronary stent μCT data and used for CFD analysis. Plotting data along reference lines in the direction of flow revealed elevated wall shear stress on stent struts, greatly reduced shear stress immediately up and downstream of struts, and recovery of shear stress in inter-strut regions. **A:** Matrix stent, with two reference lines (red and blue); **B:** Wall geometry (top) and shear stress along the upper, red reference line; **C:** Wall geometry (top) and shear stress along the lower, blue reference line. Flow from left to right, 1 Pa average wall shear stress.

Plotting shear stress along reference lines down the entire length of coronary stents revealed the impact of this disturbed flow (Fig 8). Wall shear stress on stents’ luminal surfaces was elevated. However, shear stress decreased sharply immediately up and downstream of struts. In some instances, downstream wall shear stress briefly reached negative values, indicating flow reversal. Wall shear stress began to increase further downstream from struts as the longitudinal component of velocity began to recover. The degree of this recovery, and the resultant inter-strut shear stress magnitude, gradually reduced along the length of stents. Fig 8 shows a reduction in wall shear stress of approximately 30% along a 9 mm long stent. Plotting a reference line over more complex geometry (Fig 8, blue line) showed that the presence of thin bridge struts had little additional effect.

## Discussion

We have developed a workflow that integrates particle tracking with CFD that can detect deleterious flow perturbations that are sensitive to the geometry of stents. We suggest that novel designs for stents should be routinely tested for fluid flow and shear stress effects because these are important effectors of vascular health and repair.

### Integrating particle tracking and CFD analyses

The extent to which the presence of coronary stents disturbs local fluid dynamics was revealed by tracking the motion of 10 μm particles through their structure. Flow was clearly influenced by stent struts. Streamlines were observed to follow stent struts, and were separated or funnelled by ‘peaks and valleys’ in the stent ring structure. These data suggest that particle tracking may be beneficial in predicting fluid flow in arteries modified with stents of varying geometry. The use of heat maps to display particle density goes some way to revealing this interaction. Although no relationship could be found between particle tracking and either stent strut size or orientation, this information may be revealed these experiments are replicated to allow a quantitative comparison between stent designs. CFD analysis revealed the effect of stent struts on local wall shear stress and also demonstrated the differences in fluid dynamics across dissimilar stent geometry. Wall shear stress magnitude, distribution and the extent of its reduction over stent length, were seen to vary with stent structure.

The influence of design parameters such as strut thickness and spacing have been shown in parametric studies of idealised models [23–25]. Here, it is noteworthy that particle tracking and CFD generated complementary data leading to an understanding of flow perturbation within real clinical-grade coronary stents in unprecedented detail. The fluid flow data generated by particle tracking is highly relevant to the funnelling and dispersion of cells, molecules and dissolved gases local to the arterial wall which is a key feature of vascular function. The accumulation of these bodies can promote vascular health by re-endothelialisation [7], via the capture of endothelial progenitor cells (of similar size to the fluorescent particles used here) [30], or convey deleterious effects by creating nucleation points for thrombus formation. To our knowledge, this is the first description of an in vitro model to assess particle accumulation within stented vessels.

In parallel, CFD analysis provides information on modified wall shear stress generated by coronary stents, which has long been known to adversely affect vascular health and repair. The acquisition of similar data from *in vitro* particle tracking methods, including particle image velocimetry, is extremely difficult, particularly at such high resolution. Conversely, the *in silico* simulation of the individual movement of large numbers of particles through complex stent geometry is too computationally expensive to provide information routinely on fluid flow throughout the entire lumen. Thus, the two methods generate complementary data, of relevance to healthy endothelial regrowth by both local cell migration and circulating progenitor cell capture.

Our work extends the observations of Peacock *et al* (1995) who demonstrated flow disturbances arising from the shedding of vortices from stent struts *in vitro* [31]. Benard *et al* (2003) also found areas of recirculation downstream of struts which were oriented perpendicular to flow [22]. These locations were areas of lowest shear stress, whereas regions of highest shear stress were located adjacent to struts which were parallel to flow. The impact of more complex geometry is comparable to that illustrated by idealised *in silico* modelling, which also utilises parametric studies to reveal the effect of strut dimensions, pattern and spacing on wall shear stress. Yet, idealised models lack aspects of realistic stent deployment and resultant geometry, such as strut detachment from the lumen, which can outweigh the impact of small design features. Capturing realistic geometry *in silico* has proven difficult as the radioluminescence of metallic stents renders vessel walls invisible during μCT scanning. In this respect, our study builds on the work of Migliavacca et al (2007) [32] and Gastaldi et al (2010) [33], who simulated the expansion of an idealised stent within model arteries and bifurcations, and Morlacchi et al (2011) [29], who captured patient-specific stent geometry via μCT and collapsed a simulated wall around it. By scanning and modelling a cast of the vessel lumen, we have overcome these limitations and can represent true stent and vessel wall geometry in its entirety.

### Stent design and flow

Since local haemodynamics play an important role in re-endothelialisation, they should be considered a key factor in stent design. The development of the novel model and protocol described in this work, its validation in comparison to data from established, yet less physiologically relevant parallel plate and idealised computational models, and its ability to apply various techniques to analyse a wide range of coronary stent designs contributes to that objective. In recent years, stents and secondary devices have been designed to induce favourable haemodynamic conditions. Stents which generate helical or spiral blood flow through the artery can prevent the formation of local points of low flow velocity or flow separation.

We propose that the platform that we have developed integrating particle tracking with CFD will inform and accelerate future studies in this field to design stents and other secondary implants with favourable haemodynamics. Moreover, by changing the properties of the fluid applied in these models, and taking advantage of the biocompatibility of both PDMS [34] and the closed-loop system, future studies could integrate into the platform the analysis of the influence of stents and flow on cultured endothelial cells, which could be seeded into model vessels as a bioreactor system.

### Limitations of the study

The particle tracking method was limited to two dimensions, within a volume of fluid local to the vessel wall. While this did enable the visualisation of particle motion around stents, our area of interest, it could not reveal flow perturbations further from the wall. However, CFD can complement particle tracking by providing information at fluid layers further from the vessel wall. During fluid velocity during particle tracking was restricted to a value that allowed detection of particles in multiple imaging frames, and hence this technique was carried out using a shear stress (0.1 Pa) that was lower than physiological. To address this, we performed CFD at physiological shear stress (1 Pa) which validated and extended the particle imaging analysis. The use of biologically inactive particles allowed flow to be analysed under highly controlled experimental conditions, however an inherent limitation is that biological interactions are not modelled. It would, therefore, be of interest to assess movement and deposition of biologically relevant particles, e.g. platelets, using this system.

Our model improves upon simplified and idealised models by deploying clinically approved stents, however, further work will be required to quantify flow perturbation between stent designs and to associate haemodynamically unfavourable stent design with clinical outcomes. The intention of this work is to establish a base case. We did not simulate shear stress using pulsatile flow; however the coronary artery pressure gradient is dominated by a steady contribution (we estimate flow fluctuation in the left coronary artery generates about ±18% variation in WSS) and that constant contribution is fully represented, here. On the other hand, flow fluctuations must produce time variation in the recirculation region of stent struts which, presumably, has qualitative influence upon the physiology of thrombus formation.

Further, in our CFD simulations, blood was assumed to be incompressible and Newtonian; it is in fact a high volume fraction particulate suspension (often modelled as a non-Newtonian fluid). The process of cellular margination implies the formation of a plug, with a low haematocrit region close to the wall; it is therefore possible to characterise coronary haemodynamics as multicomponent flow between two immiscible fluids, with the lumen and strut actually exposed to a Newtonian component. Nevertheless, it would be of value to simulate blood as a particulate and we have developed a single framework methodology for this [35], but the computational requirements preclude its routine use to analyse stent haemodynamics on whole vessel scales. This suggests one should assess non-Newtonian effects using a modified constitutive law with (for a fuller description) a coupled solution of species convection-diffusion, to account for the transport of haematocrit [36]. Though very computationally expensive, this is achievable in principle, even in the presence of complex stent geometry.

Moreover, the straight, uniform model vessels did not reflect typical anatomical geometry, nor were their PDMS walls thin enough for that geometry to respond to pulsatile flow. Therefore, there is scope for technical refinement of our models by modifying the relevant fabrication, experimental, or simulation protocols.

## Supporting information

S1 Fig Assessing stent geometry. The geometry of deployed stents was assessed by measuring strut dimensions, and calculating the metal-to-artery ratio and strut orientation. Top: Representative image of a deployed coronary stent as they appeared under magnification, along its longitudinal axis. Bottom: Strut orientation was calculated by measuring the angle of struts relative to flow (left) and the length of struts at each orientation as a percentage of the total (right).
(TIF)

S2 Fig *In vitro* model fabrication and stent deployment. Top: To create model vessels, PDMS was poured into a mould (left). Once cured, the mould (consisting of a cuvette, rod and rubber caps) was removed, leaving the resultant PDMS model vessel (right). Bottom: Coronary stents were deployed in the model via the inflation of balloon catheters.
(TIF)

S3 Fig Particle tracking protocol. (A) Video recordings were taken at overlapping fields of view along the total length of stents. (B) Two image stacks, each representing 30 seconds of flow, were separated from individual recordings at each field of view. (C) Flowing particles were tracked in each stack, and local stent geometry was isolated. (D) Particle tracks and stent geometry were combined, to visualise local flow patterns. (E) Heat maps of particle distribution were created by counting tracks, and the number of particles moving along them, in relation to a 2.5 mm x 1.5 mm grid of 100 μm squares.
(TIF)

S4 Fig Representative particle tracking data. A particle suspension was pumped through an empty model vessel. A video recording was taken, from which individual particle motion was tracked. **Top:** Tracking revealed particles following streamlines (red: 1 particle per streamline, green: 2, blue: 3). **Bottom:** Heat map of particle density, illustrating the location of streamlines and the frequency of particles moving along them over a superimposed 100 μm grid. Flow from left to right, 12.8 ml/min.
(TIF)

S5 Fig PDMS casts of coronary stents. PDMS was cured within the lumen of model vessels containing coronary stents, which were then removed to create casts for μCT scanning. Casting captured clean, well-defined struts even within complex geometry (red boxes). **Left:** Cast of a Pro Kinetic Energy coronary stent. **Right:** Cast of a Velocity coronary stent, 5:1 ratio. Scale bar: 0.5 mm.
(TIF)

S6 Fig Coronary stent strut orientation. The orientation of coronary stent struts was measured in relation to the direction of applied flow (0°), as illustrated in S2 Fig. The length of struts at each angle (to the nearest 10°) is shown here as a percentage of the total strut length. Each chart represents an average measurement taken from two faces of each stent (rotating the stent 90° between the two) and one repeating unit of stent geometry.
(TIF)

S7 Fig Tracked particle density heat maps in BiodivYsio OC and Chroma coronary stents. Particles were tracked moving through coronary stents deployed within model vessels. The position of streamlines and the frequency of particles tracked moving along them was measured in relation to a 100 μm square grid and presented as a percentage of the total number of particles seen in each grid. **Left:** BiodivYsio OC stent. **Right:** Chroma stent. Flow from bottom to top, Re = 68 (equivalent to blood flow with 1 Pa wall shear stress). The plane of focus is on the bottom of the vessel.
(TIF)

S8 Fig Particle track orientation and coronary stent geometry. The orientation of tracked particle streamlines moving through coronary stents deployed within model vessels was measured to quantify the impact of stent design on the direction of flow and, therefore, the direction of mechanical cues cells would be exposed to. The length of tracks at an angle greater than ±5° of the direction of flow (0°), as a percentage of the total length of tracks within each stent, is shown against stent geometry. There is no clear relationship between stent strut angle or strut thickness and the deviation of flow. **Top:** Track orientation and the mode angle of coronary stent struts relative to flow. **Bottom:** Track orientation and coronary stent strut thickness.
(TIF)

S9 Fig Convergent results from particle imaging and CFD. (A) *In silico* models were reconstructed from Coroflex Blue stent strut μCT data and used for CFD analysis. Velocity fields are depicted at the vessel wall, with the length of each arrow being proportional to velocity. (B) Coroflex Blue stents were deployed in PDMS model vessels and particle tracking was performed for two 30-second-long sequences (red and blue tracks). Re = 68 (equivalent to blood flow with 1 Pa wall shear stress). (A, B) Particle tracking and CFD showed convergence with funnelling of the flow towards strut features.
(TIF)

S1 Table (DOCX)

S2 Table (DOCX)
