# Correction: Integrating particle tracking with computational fluid dynamics to assess haemodynamic perturbation by coronary artery stents

**DOI:** 10.1371/journal.pone.0314392

**Published:** 2024-11-19

**Authors:** Luke Boldock, Amanda Inzoli, Silvia Bonardelli, Sarah Hsiao, Alberto Marzo, Andrew Narracott, Julian Gunn, Gabriele Dubini, Claudio Chiastra, Ian Halliday, Paul D. Morris, Paul C. Evans, Perrault C. M.

The affiliation for the eighth author is incorrect. Gabriele Dubini is not affiliated with #5 but with #3: Laboratory of Biological Structure Mechanics–LaBS, Department of Chemistry, Materials and Chemical Engineering ‘Giulio Natta’, Politecnico di Milano, Milan, Italy.
