# Supplementary figures and images for: Integrating particle tracking with computational fluid dynamics to assess haemodynamic perturbation by coronary artery stents
Source: PLoS One. 2022 Jul 28;17(7):e0271469. doi: 10.1371/journal.pone.0271469 (PMC9333229; doi:10.1371/journal.pone.0271469)

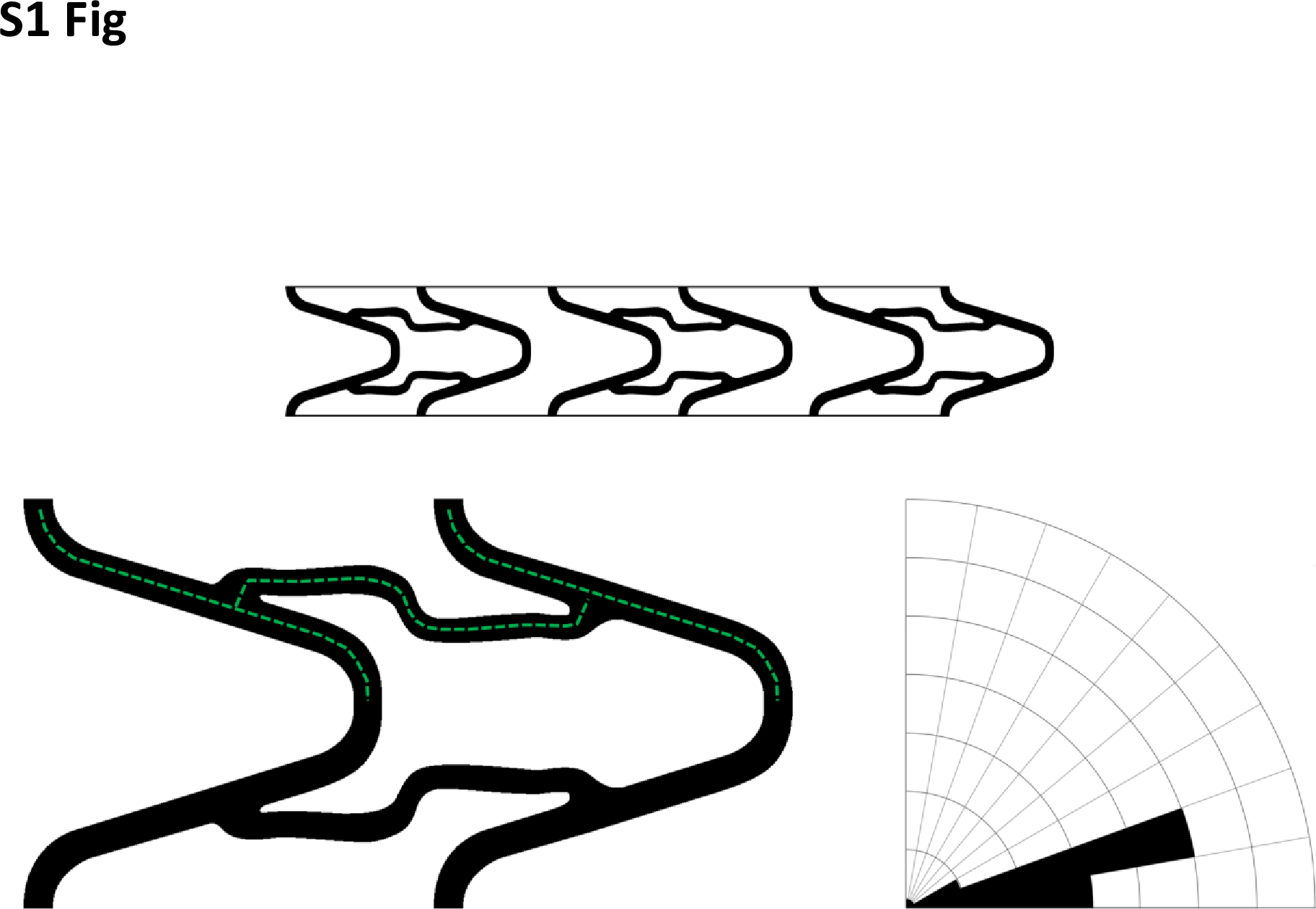

Supplement: S1 Fig — The geometry of deployed stents was assessed by measuring strut dimensions, and calculating the metal-to-artery ratio and strut orientation. Top: Representative image of a deployed coronary stent as they appeared under magnification, along its longitudinal axis. Bottom: Strut orientation was calculated by measuring the angle of struts relative to flow (left) and the length of struts at each orientation as a percentage of the total (right). (TIF) [file pone.0271469.s001.tif]

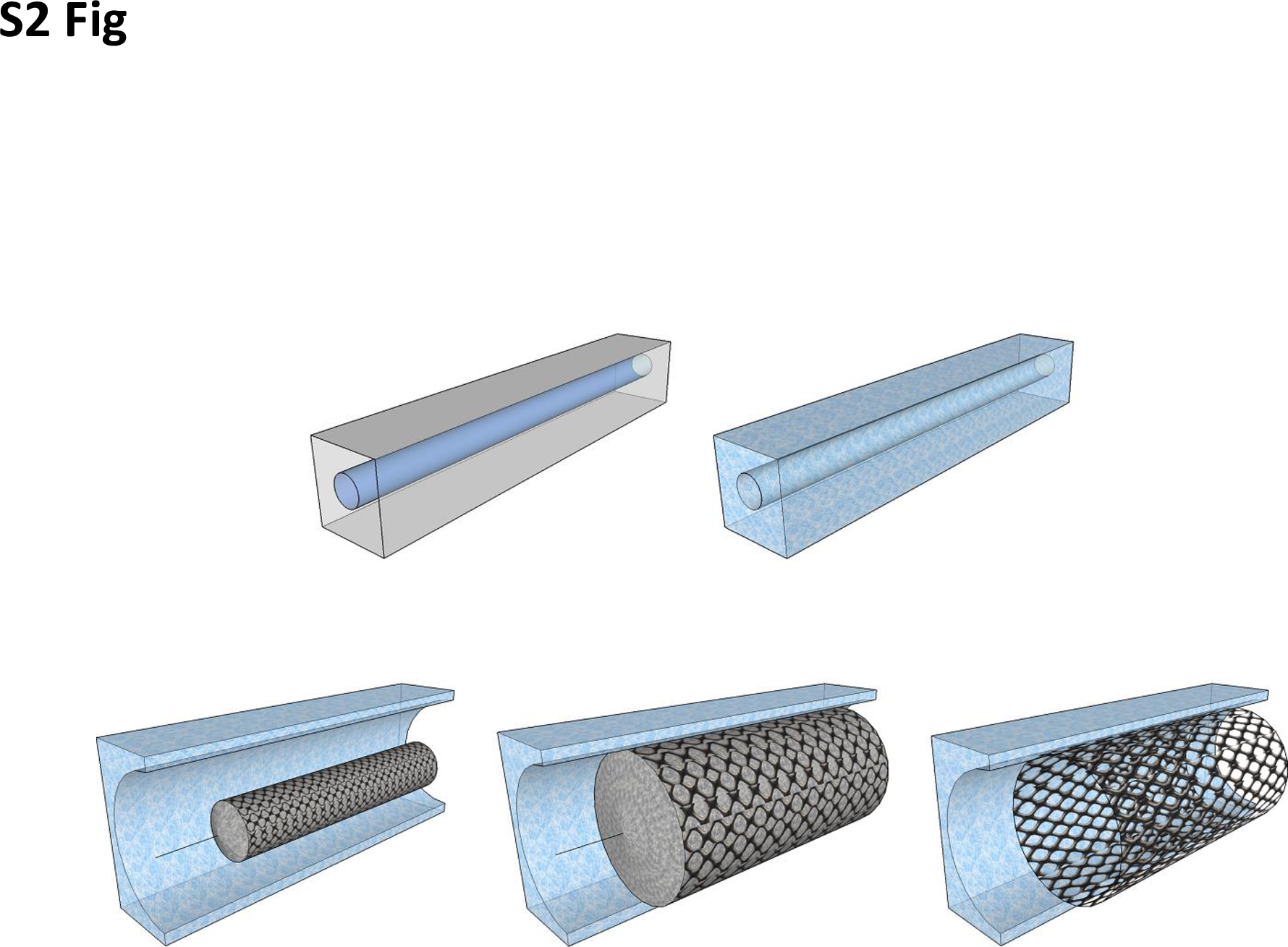

Supplement: S2 Fig — Top: To create model vessels, PDMS was poured into a mould (left). Once cured, the mould (consisting of a cuvette, rod and rubber caps) was removed, leaving the resultant PDMS model vessel (right). Bottom: Coronary stents were deployed in the model via the inflation of balloon catheters. (TIF) [file pone.0271469.s002.tif]

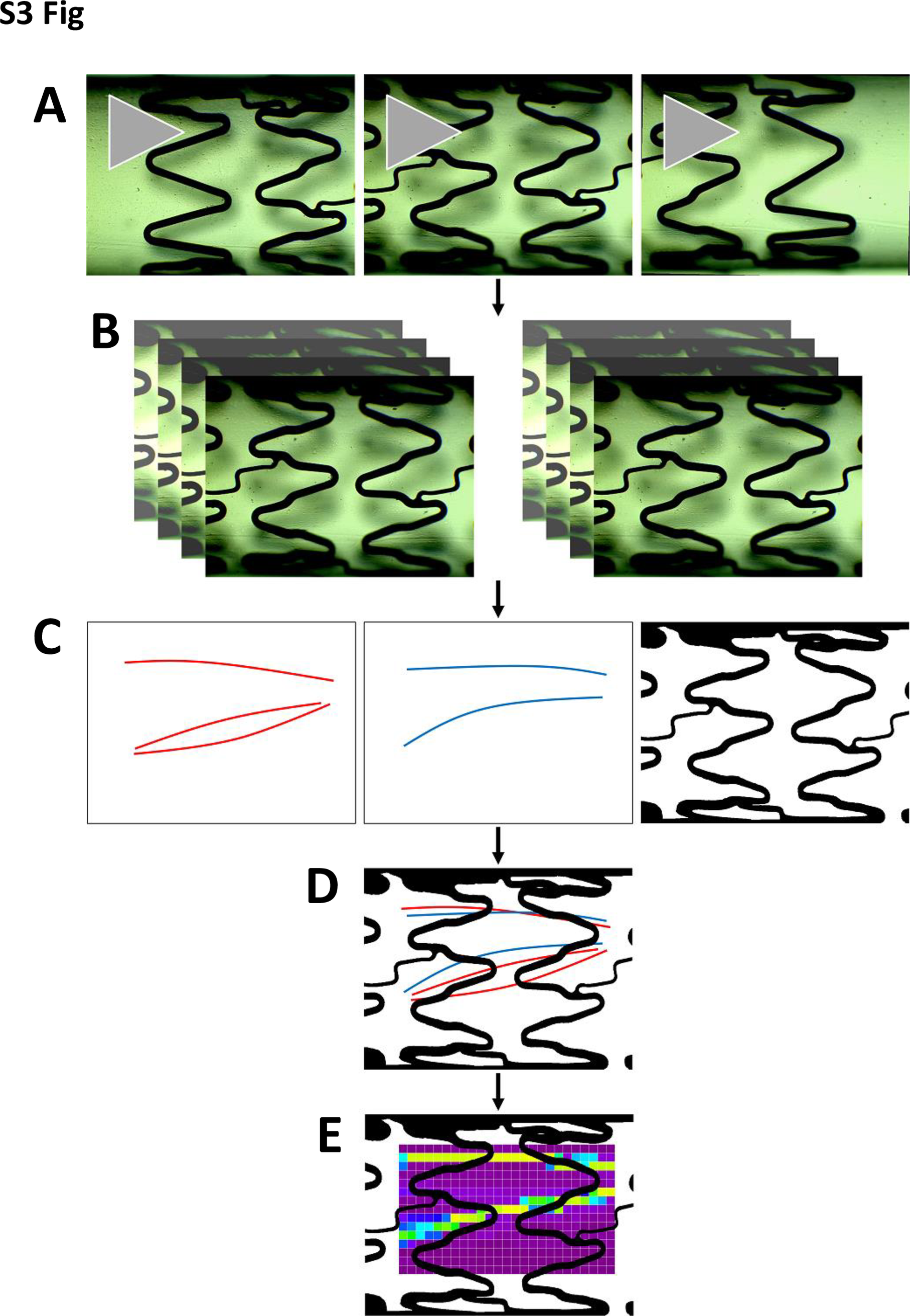

Supplement: S3 Fig — (A) Video recordings were taken at overlapping fields of view along the total length of stents. (B) Two image stacks, each representing 30 seconds of flow, were separated from individual recordings at each field of view. (C) Flowing particles were tracked in each stack, and local stent geometry was isolated. (D) Particle tracks and stent geometry were combined, to visualise local flow patterns. (E) Heat maps of particle distribution were created by counting tracks, and the number of particles moving along them, in relation to a 2.5 mm x 1.5 mm grid of 100 μm squares. (TIF) [file pone.0271469.s003.tif]

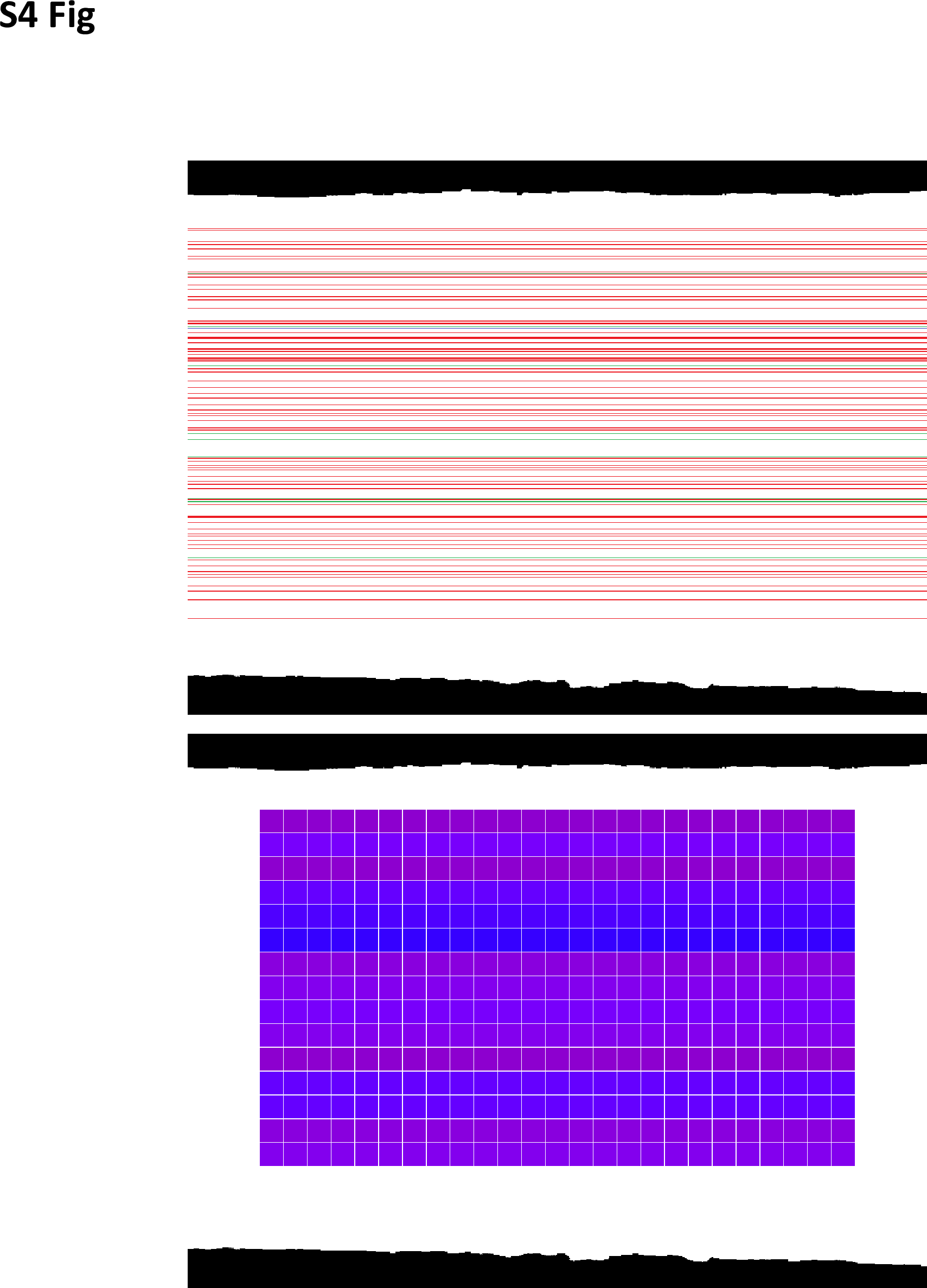

Supplement: S4 Fig — A particle suspension was pumped through an empty model vessel. A video recording was taken, from which individual particle motion was tracked. Top: Tracking revealed particles following streamlines (red: 1 particle per streamline, green: 2, blue: 3). Bottom: Heat map of particle density, illustrating the location of streamlines and the frequency of particles moving along them over a superimposed 100 μm grid. Flow from left to right, 12.8 ml/min. (TIF) [file pone.0271469.s004.tif]

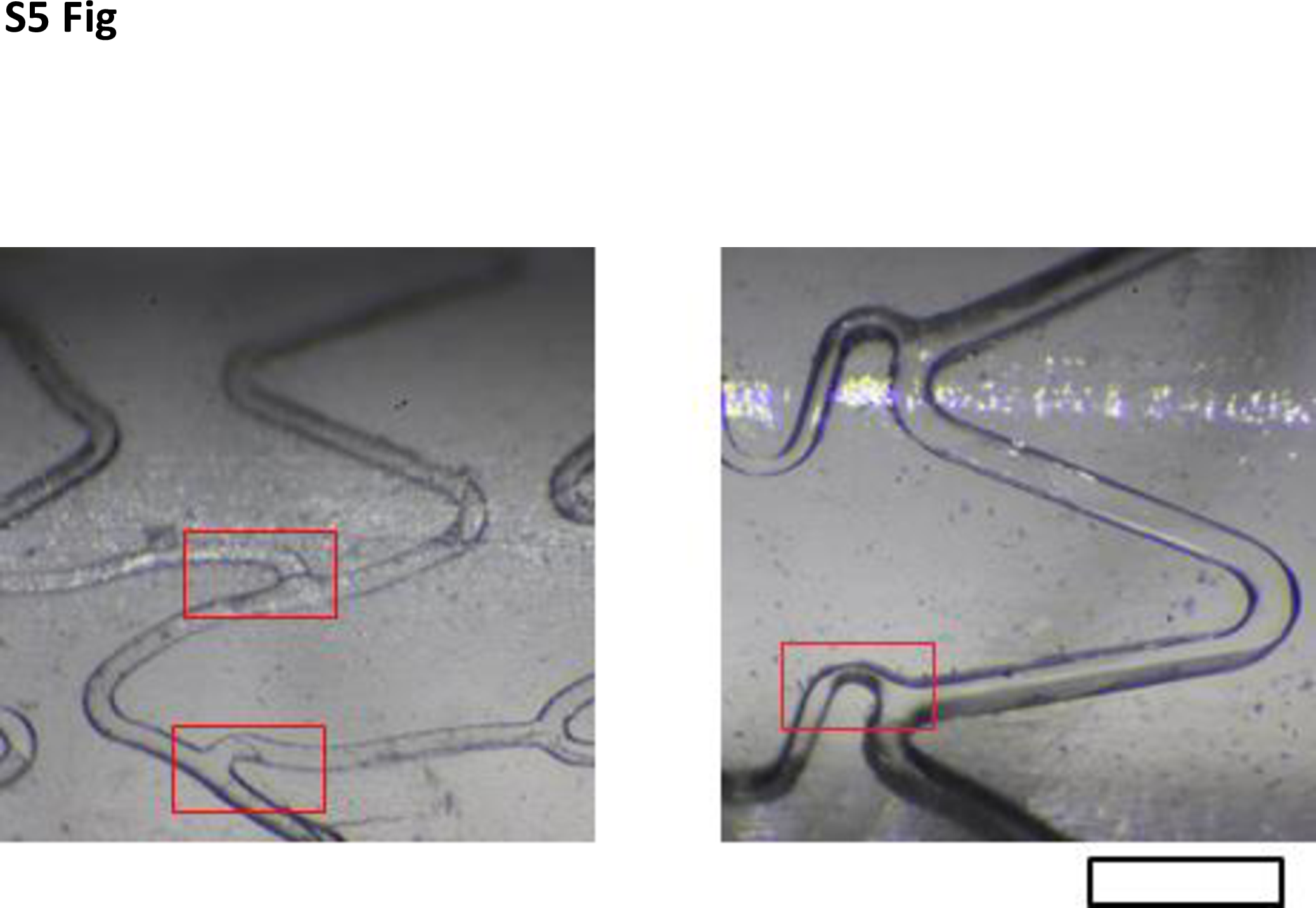

Supplement: S5 Fig — PDMS was cured within the lumen of model vessels containing coronary stents, which were then removed to create casts for μCT scanning. Casting captured clean, well-defined struts even within complex geometry (red boxes). Left: Cast of a Pro Kinetic Energy coronary stent. Right: Cast of a Velocity coronary stent, 5:1 ratio. Scale bar: 0.5 mm. (TIF) [file pone.0271469.s005.tif]

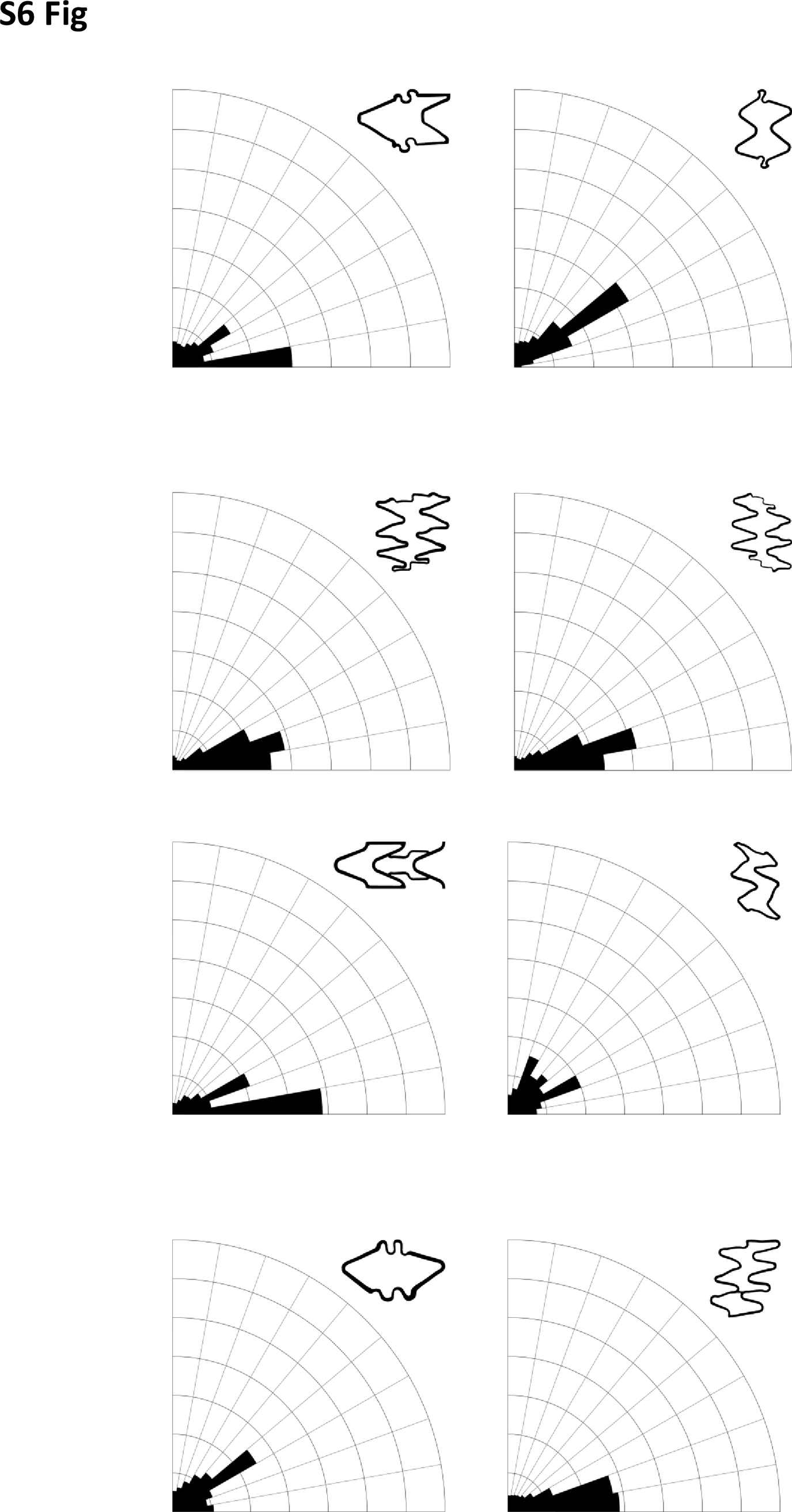

Supplement: S6 Fig — The orientation of coronary stent struts was measured in relation to the direction of applied flow (0°), as illustrated in S2 Fig. The length of struts at each angle (to the nearest 10°) is shown here as a percentage of the total strut length. Each chart represents an average measurement taken from two faces of each stent (rotating the stent 90° between the two) and one repeating unit of stent geometry. (TIF) [file pone.0271469.s006.tif]

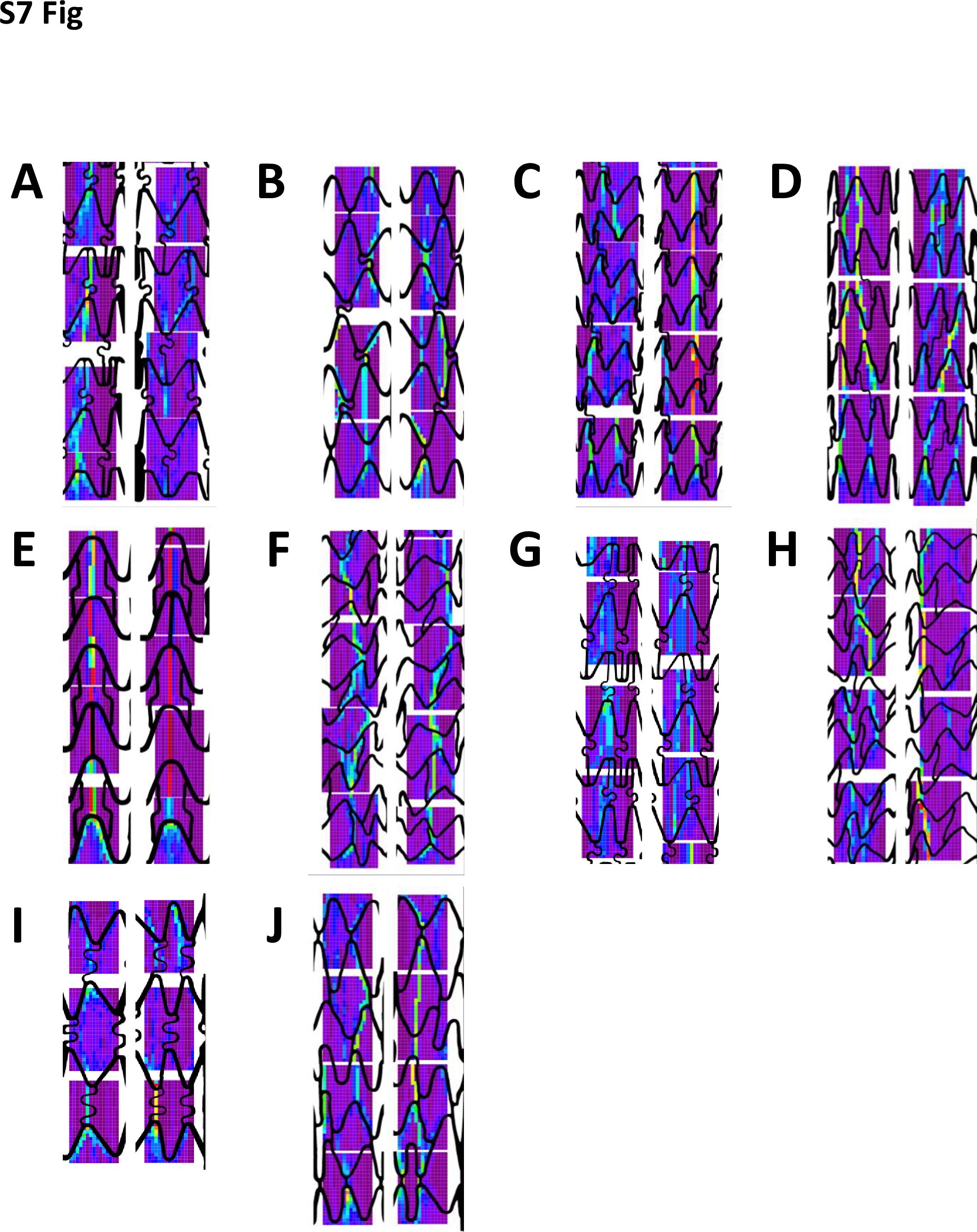

Supplement: S7 Fig — Particles were tracked moving through coronary stents deployed within model vessels. The position of streamlines and the frequency of particles tracked moving along them was measured in relation to a 100 μm square grid and presented as a percentage of the total number of particles seen in each grid. Left: BiodivYsio OC stent. Right: Chroma stent. Flow from bottom to top, Re = 68 (equivalent to blood flow with 1 Pa wall shear stress). The plane of focus is on the bottom of the vessel. (TIF) [file pone.0271469.s007.tif]

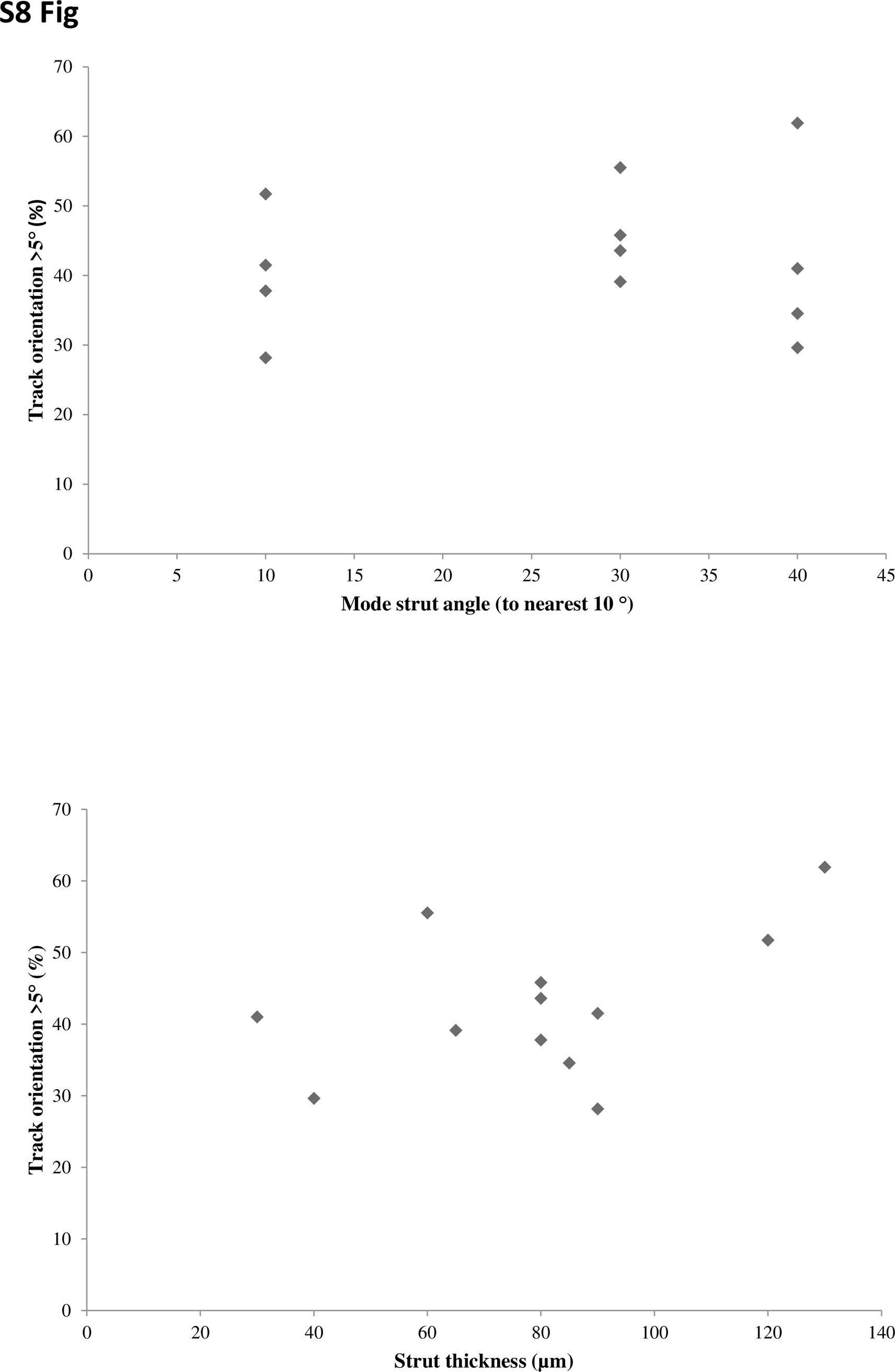

Supplement: S8 Fig — The orientation of tracked particle streamlines moving through coronary stents deployed within model vessels was measured to quantify the impact of stent design on the direction of flow and, therefore, the direction of mechanical cues cells would be exposed to. The length of tracks at an angle greater than ±5° of the direction of flow (0°), as a percentage of the total length of tracks within each stent, is shown against stent geometry. There is no clear relationship between stent strut angle or strut thickness and the deviation of flow. Top: Track orientation and the mode angle of coronary stent struts relative to flow. Bottom: Track orientation and coronary stent strut thickness. (TIF) [file pone.0271469.s008.tif]

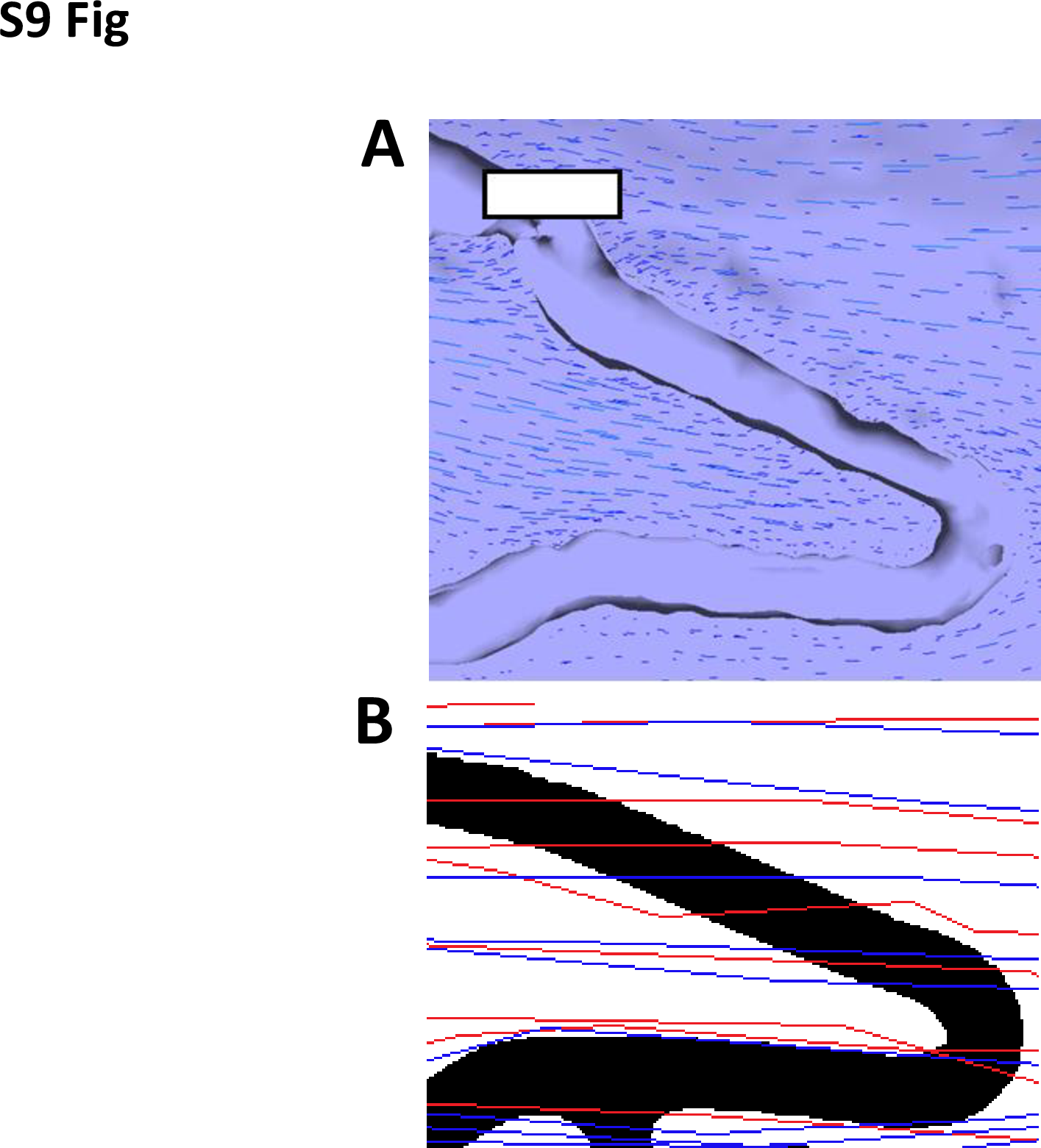

Supplement: S9 Fig — (A) In silico models were reconstructed from Coroflex Blue stent strut μCT data and used for CFD analysis. Velocity fields are depicted at the vessel wall, with the length of each arrow being proportional to velocity. (B) Coroflex Blue stents were deployed in PDMS model vessels and particle tracking was performed for two 30-second-long sequences (red and blue tracks). Re = 68 (equivalent to blood flow with 1 Pa wall shear stress). (A, B) Particle tracking and CFD showed convergence with funnelling of the flow towards strut features. (TIF) [file pone.0271469.s009.tif]
