# Supplementary material for: Integrating particle tracking with computational fluid dynamics to assess haemodynamic perturbation by coronary artery stents
Source: PLoS One. 2022 Jul 28;17(7):e0271469. doi: 10.1371/journal.pone.0271469 (PMC9333229; doi:10.1371/journal.pone.0271469)
Supplement: S1 Table — (DOCX) [file pone.0271469.s010.docx]

| **Coating** | Phosphorylcholine | None | None | None | None |
| --- | --- | --- | --- | --- | --- |
| **Material** | 316L stainless steel | MP35N cobalt chromium | L605 cobalt chromium | L605 cobalt chromium | 316L stainless steel |
| **Unit (not to scale)** | 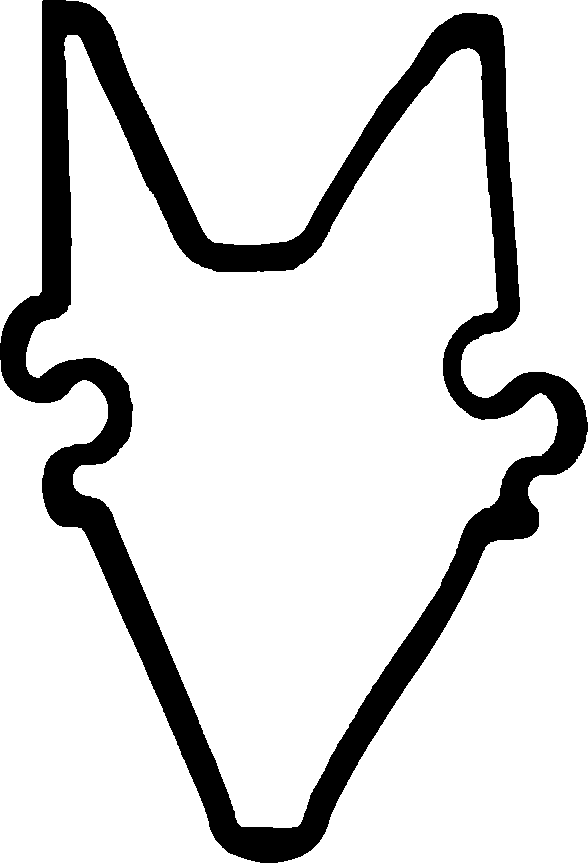 | 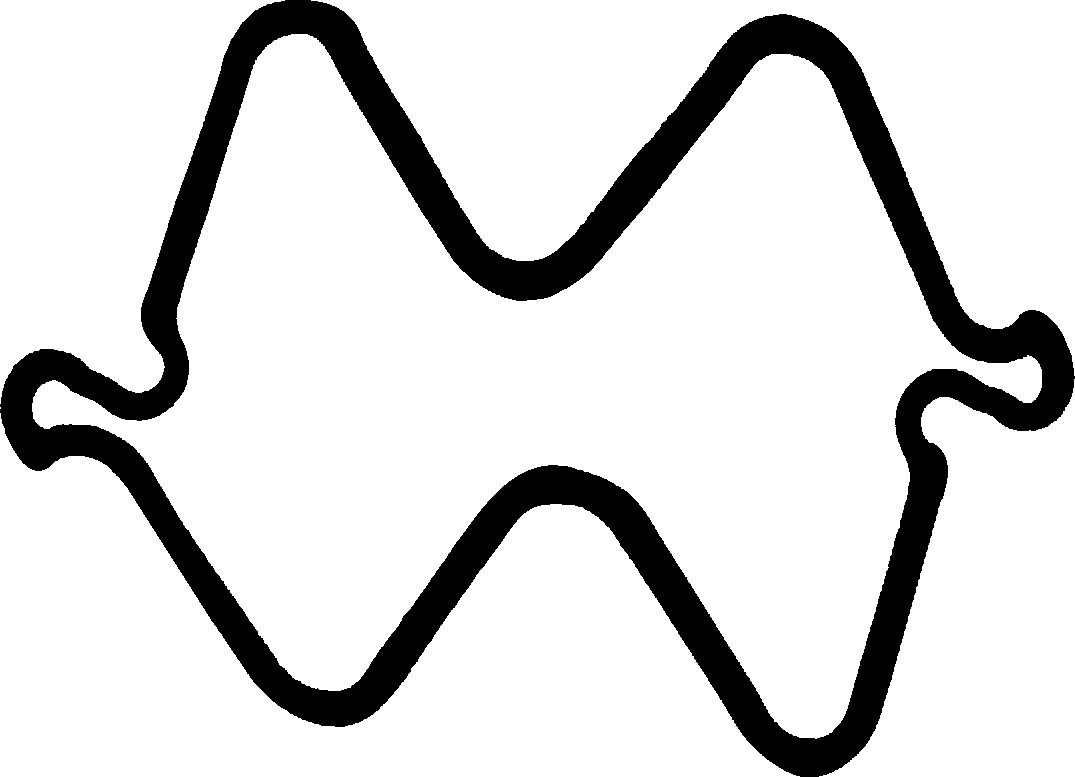 | 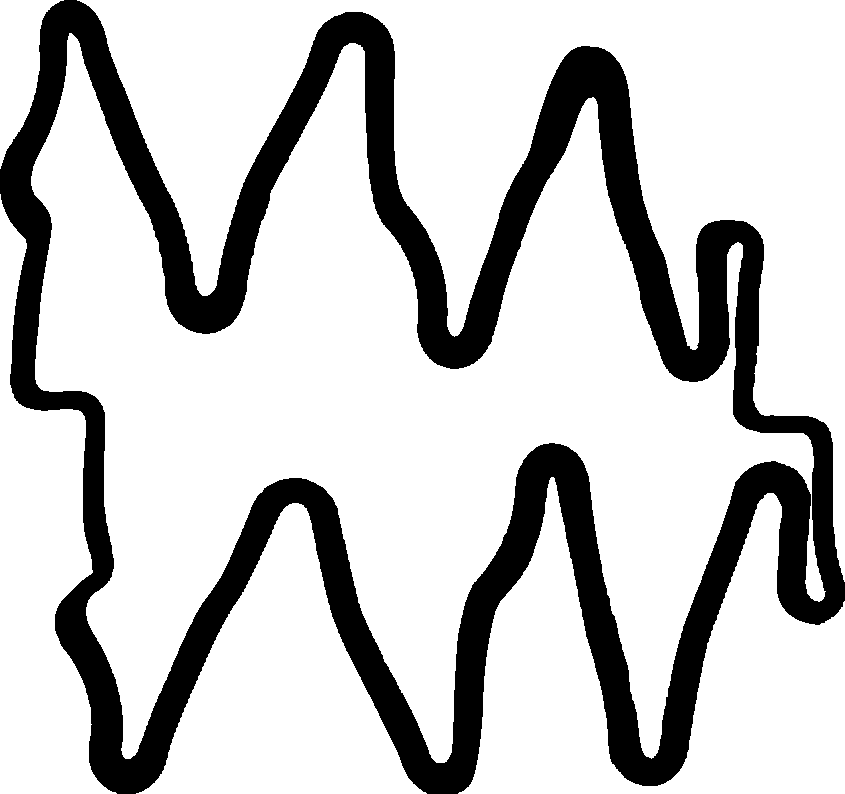 | 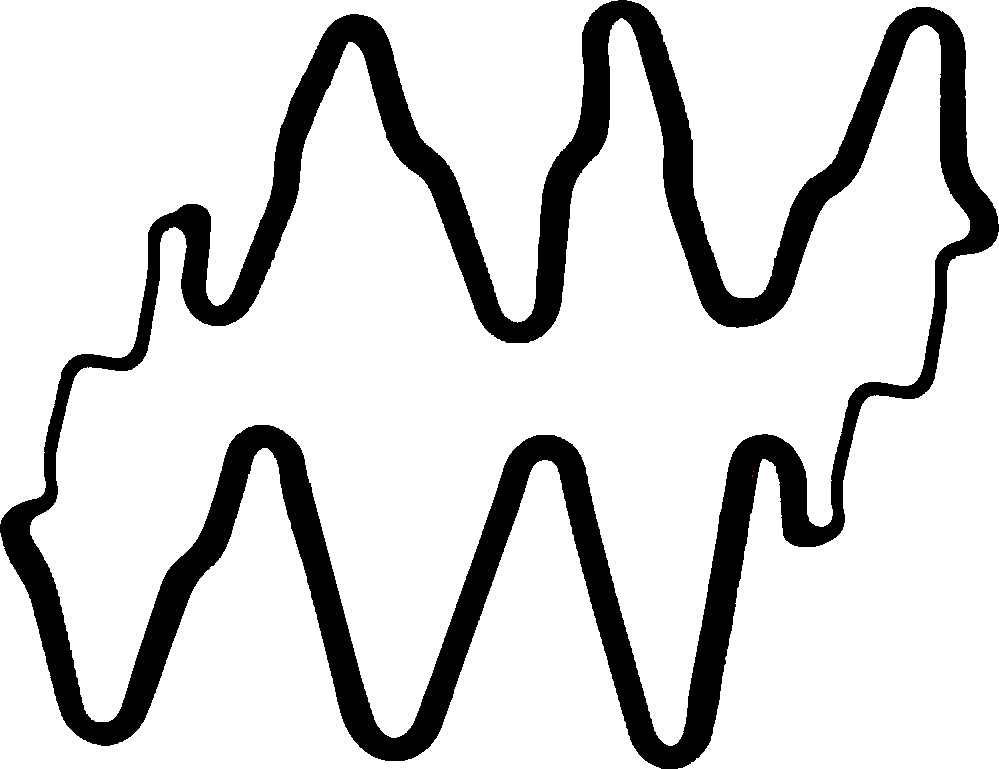 | 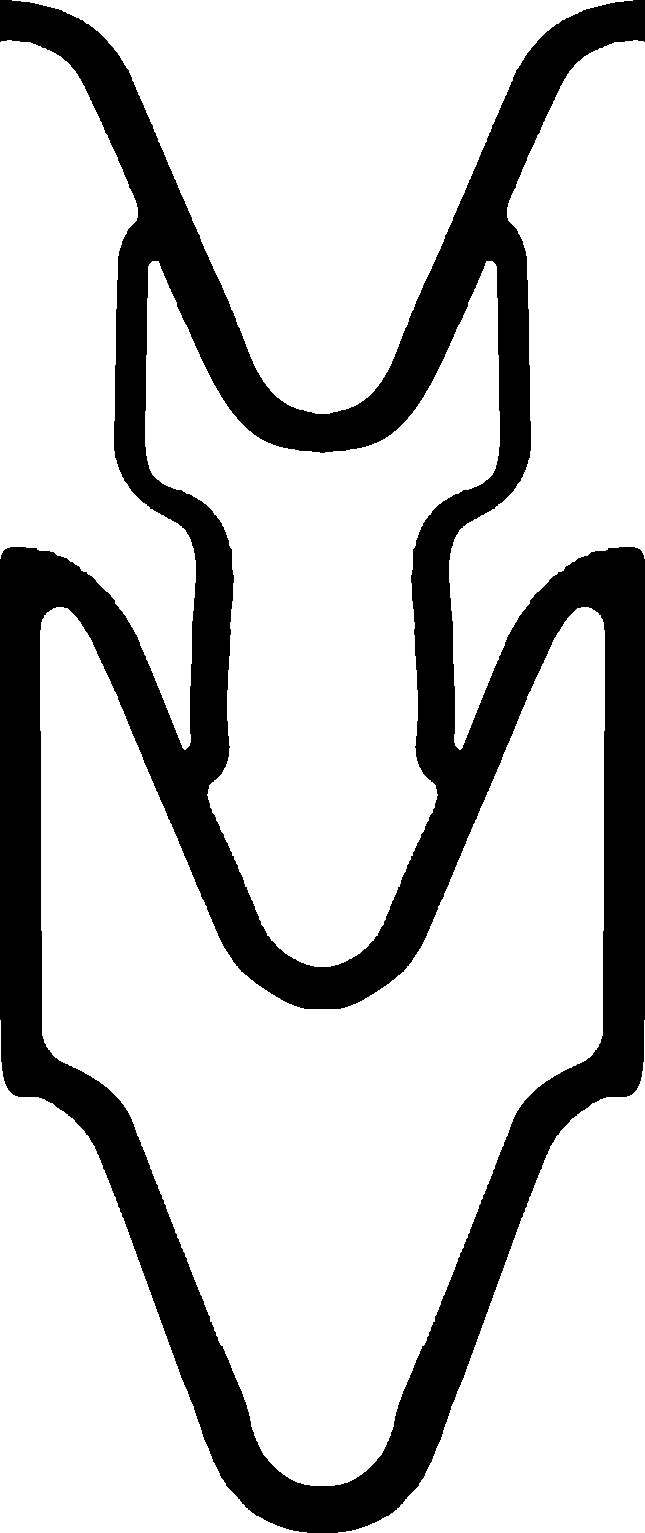 |
| **MSA (%)** | 17.6 | 16.7 | 23.1 | 18.8 | 24.2 |
| **Strut (µm)** | 90 | 85 | 65 | 60 | 80 |
| **Manufacturer** | Biocompatibles Ltd. | Biosensors International | B Braun Medical Inc. | B Braun Medical Inc. | Sahajanand Medical Technologies |
| **Model** | BiodivYsio OC | Chroma | Coroflex Blue | Coroflex Blue Neo | Matrix |

Table A Properties of coronary stents
