# Supplementary material for: Integrating particle tracking with computational fluid dynamics to assess haemodynamic perturbation by coronary artery stents
Source: PLoS One. 2022 Jul 28;17(7):e0271469. doi: 10.1371/journal.pone.0271469 (PMC9333229; doi:10.1371/journal.pone.0271469)
Supplement: S2 Table — (DOCX) [file pone.0271469.s011.docx]

| **Coating** | Silicon carbide, sirolimus in polylactide matrix | Phosphorylcholine | Silicon carbide | None | None |
| --- | --- | --- | --- | --- | --- |
| **Material** | L605 cobalt chromium | 316L stainless steel | L605 cobalt chromium | 316L stainless steel | F562 cobalt chromium |
| **Unit** | 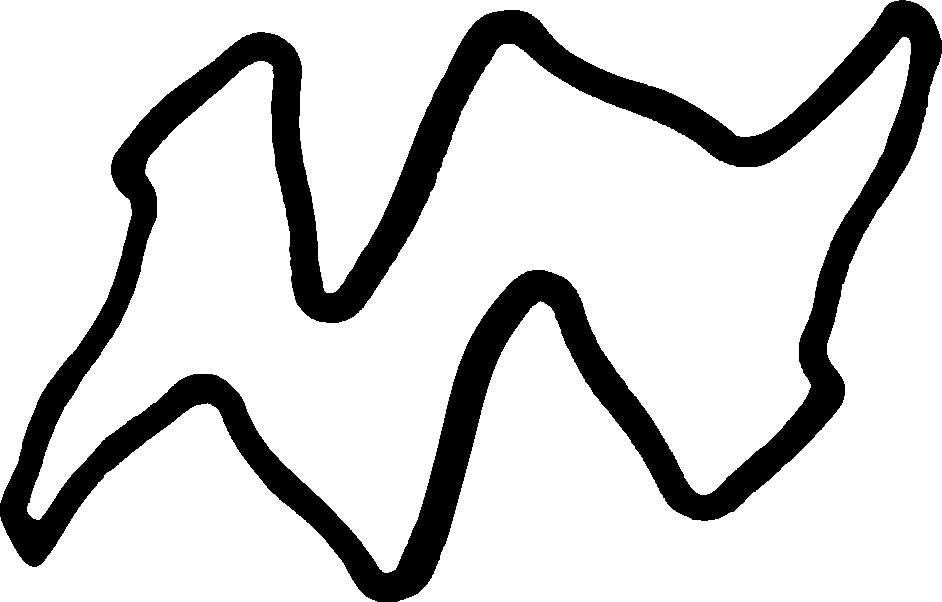 | 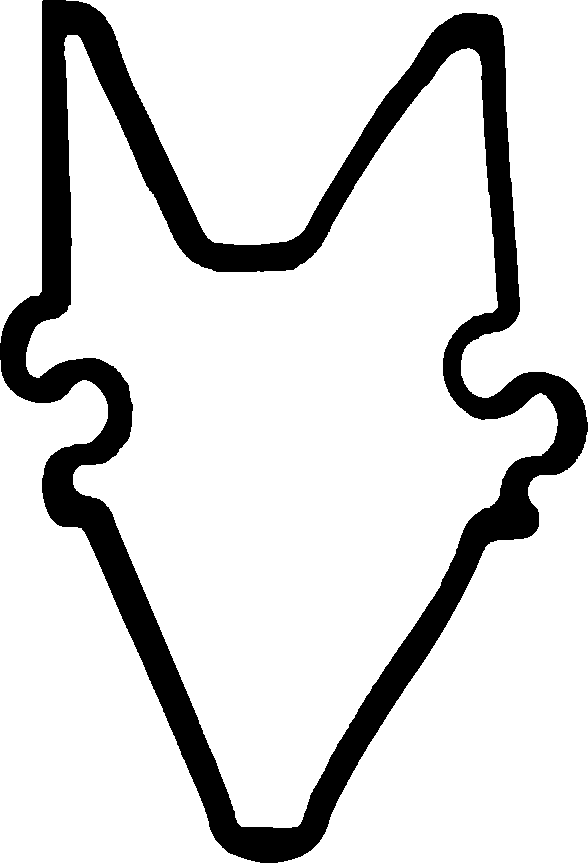 | 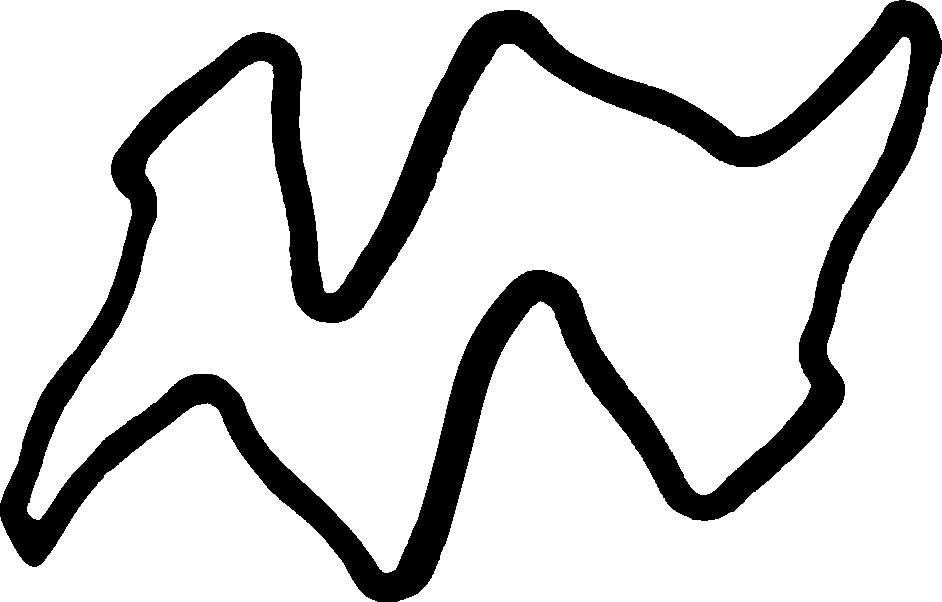 | 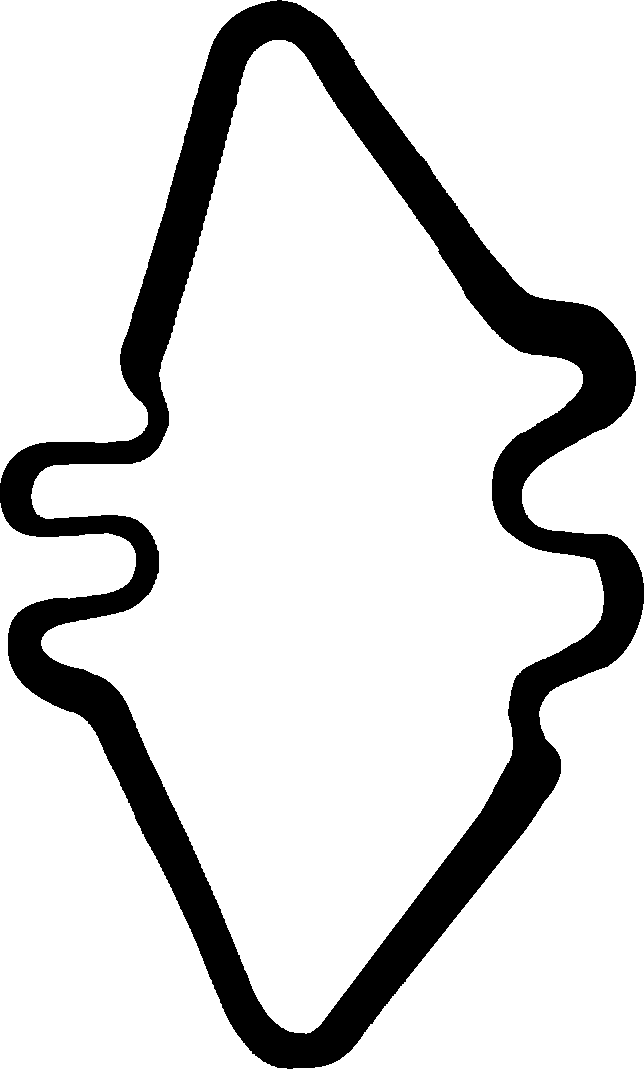 | 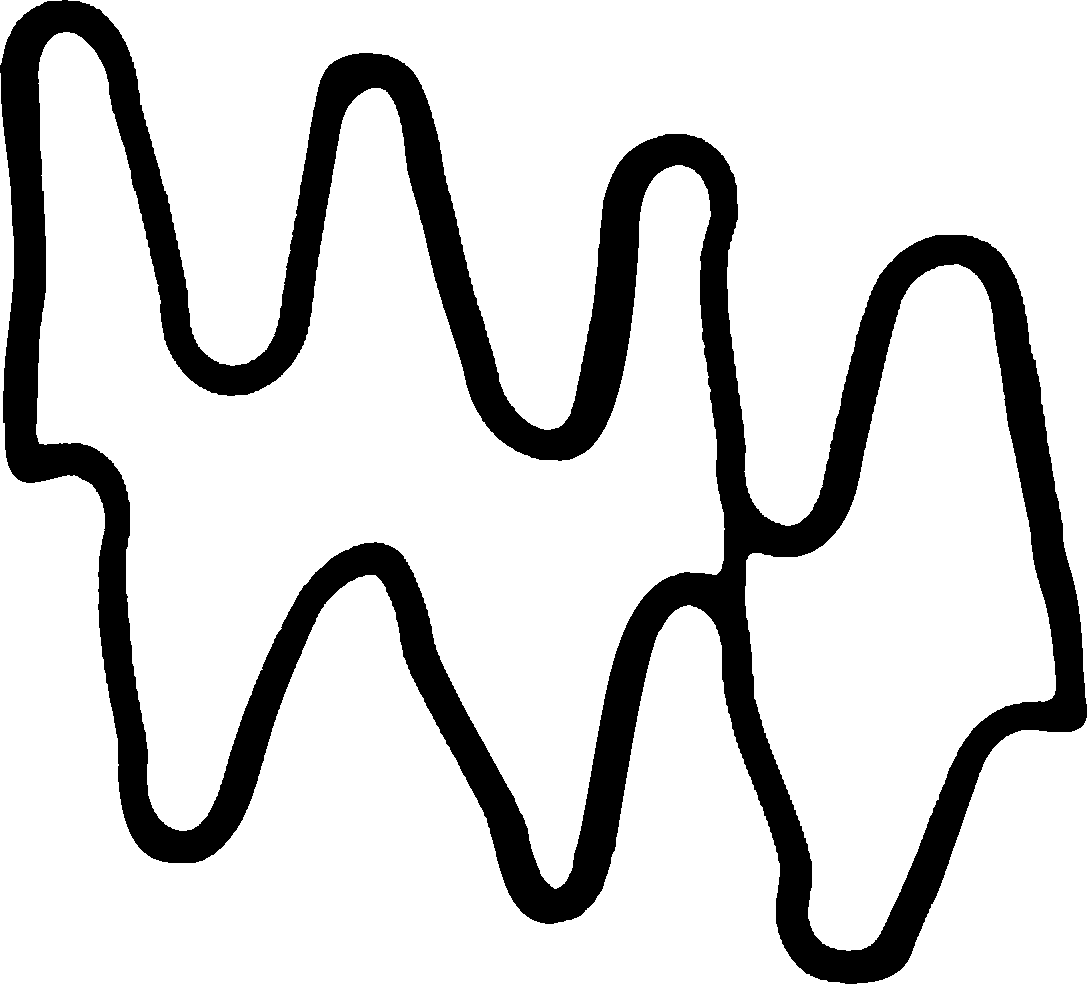 |
| **MSA (%)** | 18.5 | 17.6 | 18.5 | 17.9 | 18.7 |
| **Strut (µm)** | 60/80 | 90 | 60/80 | 130 | 120 |
| **Manufacturer** | Biotronik | Biocompatibles | Biotronik | Cordis | Blue Medical |
| **Model** | Orsiro | Penchant | Pro Kinetic Energy | Velocity | XTRM Track |

Table B Properties of coronary stents
